# Reinforcement Learning-Based Temperature Control of Lab-Scale Continuous Stirred Tank Reactor

**DOI:** 10.1021/acsomega.6c04451

**Published:** 2026-08-18

**Authors:** Jim George, Anagha Ravikumar, Erin Joshy, Alin Cheriyan, Thirunavukkarasu Indiran

**Affiliations:** † Department of Electrical and Electronics Engineering, 585197 Muthoot Institute of Technology and Science, Ernakulam, Kerala 682038, India; ‡ 76793 Manipal Institute of Technology, Manipal Academy of Higher Education, Manipal, Karnataka 576104, India

## Abstract

Continuous Stirred Tank Reactors (CSTRs) are widely used in chemical process industries, where precise temperature control is crucial for product quality, operational effectiveness, and safety. However, the accuracy of strict first-principle models is limited by the extremely nonlinear thermal behavior brought on by Arrhenius-based kinetics, measurement noise, transport delay, and feed fluctuations. In order to overcome this, the current study uses multivariate pilot-plant time-series data to create a high-fidelity nonlinear autoregressive model with exogenous inputs (NARX). Twin Delayed Deep Deterministic Policy Gradient (TD3) and Soft Actor-Critic (SAC) are used in the NARX model as a reinforcement learning environment for closed-loop temperature regulation. Feed temperatures, feed flow rates, and hot-oil-bath actuation are among the various manipulated-variable combinations that are assessed. The suggested NARX–RL framework effectively tracks set points, according to the results. Both SAC and TD3 exhibit acceptable closed-loop performance, according to a comparative analysis; however, TD3 consistently outperforms SAC in tracking indices, convergence speed, and control stability across the majority of manipulated-variable combinations. Overall, the proposed NARX-based reinforcement learning technique provides a feasible option for flexible, data-driven, and practically implementable temperature regulation in lab-scale CSTR systems.

## Graphical abstract

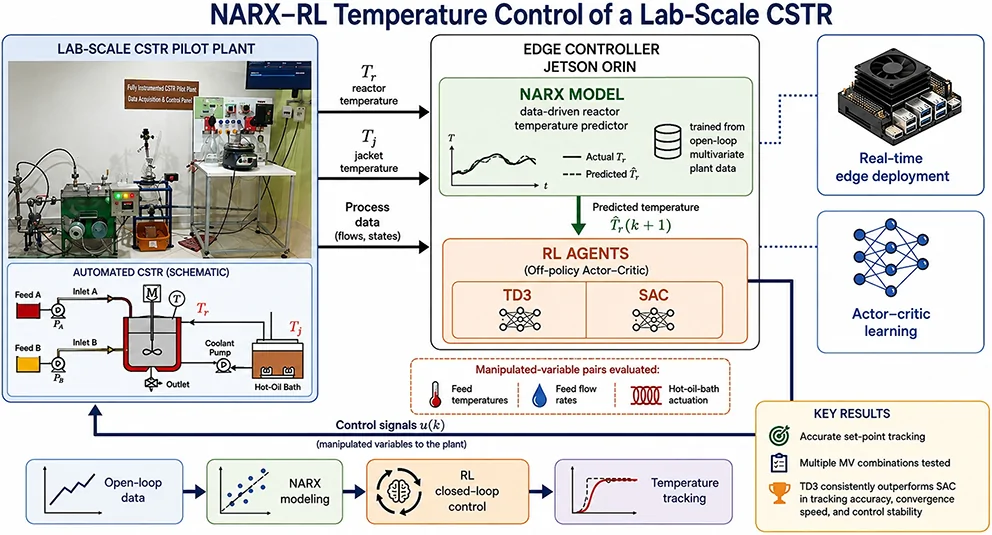

## 1 Introduction

A Continuous Stirred Tank Reactor (CSTR) is widely used in chemical and process industries because it supports controlled operation and continuous production through the continuous feeding, stirring, and processing of reactants. Its agitator promotes uniform temperature and concentration throughout the reactor volume, making the CSTR suitable for monitoring, analysis, and control.

### 1.1 Model-Based Control Strategies for Continuous Stirred Tank Reactors

Nonlinear model-based control techniques for CSTR systems with an emphasis on precise output tracking under process uncertainty are dealt in refs 1,19 . Reference^1^ integrates a first-principle nonlinear model with an adaptive fuzzy backstepping controller based on the Barrier Lyapunov Function,using temperature and concentration as major states and coolant temperature as the controlled input. Reference^19^ develops a neural-network-based adaptive backstepping controller with covariance resetting for a nonlinear, nonaffine CSTR, achieving improved concentration tracking with reduced oscillations and control effort under parameter uncertainty. Although the approach strengthens robustness when process data are limited, its simulation-based validation leaves opportunities for experimental testing, multivariable extension, actuator-saturation handling, and unmeasurable-state estimation. References 20,26,28,31 mostly look on structured model-based control techniques for reactor temperature regulation and disturbance rejection. Reference^20^ suggests an iterative-learning-observer-based predefined-time sliding mode controller that handles input-output limitations and disturbances while guaranteeing designer-specified convergence time. Reference^26^, in comparison to traditional PID and linear-surface DSMC techniques, empirically demonstrated a hybrid Dynamic Sliding Mode Control framework with a nonlinear PID sliding surface reduces integral square error (ISE), overshoot, settling time, and chattering. The study^28^ combines dynamic matrix control, PID cascade control, Smith prediction, and a disturbance observer to enhance reactor temperature regulation under lag, nonlinearity, and disturbances. References 22,24,31 advance CSTR control toward optimization and uncertainty-aware operation through gain-scheduled mean-field-type control, optimal switched-system control, and metaheuristic state-dependent-parameter proportional-integral-plus control, respectively. In general, references 20,22,24,26,28,31 demonstrate how model-based CSTR control has progressed from deterministic nonlinear regulation to risk-aware control under uncertainty, optimal switching, and structured temperature management.

### 1.2 Reinforcement Learning and Intelligent Control Strategies for CSTRs

According to ref^2^, adaptive critic designs, reinforcement learning (RL), and nonlinear Model Predictive Control (NMPC) provide promising solutions for managing the nonlinear dynamics of Continuous Stirred Tank Reactors (CSTRs). However, practical implementations must account for nonzero equilibrium conditions, realistic operating constraints, dynamic trajectory tracking, asymmetric input limits, decentralized cascade-reactor control, and reduced real-time computational burden, as emphasized in ref^4^. Thus, references 2,4 establish RL, adaptive critic designs, and nonlinear optimal control as significant directions for flexible and scalable CSTR control. The work^7^ integrates adaptive control for nonlinear process dynamics with reinforcement learning to provide a timely and pertinent contribution to CSTR/CSTH temperature regulation. Although both TD3 and SAC are appropriate for continuous control, the given results indicate that TD3 with a Gaussian reward function performs better in tracking and disturbance-rejection than SAC in the heating process under study. Reference^33^ suggested for highly nonlinear semiconductor manufacturing process impacted by drift, noise, and dynamic operating fluctuations, Soft Actor–Critic-based reinforcement learning controller. The study’s model-free adaptive control philosophy is very applicable to CSTR applications, where nonlinear kinetics, disturbances, and uncertainty frequently diminish the efficacy of traditional model-based controllers, even if it is not specifically focused on CSTR temperature regulation. In situations where precise first-principles modeling is challenging, the work encourages the application of SAC-family reinforcement learning techniques to nonlinear reactor systems like CSTRs. A thorough analysis of reinforcement learning applications in low-level control, controller tuning, optimization, fault detection, and fault-tolerant control was provided by ref^34^. The review is highly relevant to CSTR temperature control because it highlights the suitability of RL for nonlinear, uncertain, and continuously operated process systems where conventional model-based controllers may require accurate models and frequent retuning. The study encourages adaptive temperature regulation solutions based on TD3, SAC, and related actor-critic techniques by supporting the usage of RL-based control and optimization frameworks for handling manipulated variables like reactant flow rate and heat input in the context of CSTRs. Reference^35^ presented a distributed trading control framework based on Soft Actor–Critic for real-time optimization of active distribution networks under operational restrictions and unknown operating conditions. The work is pertinent to CSTR temperature regulation because it shows the efficacy of SAC for nonlinear, limited, and continuous decision-making issues, even if the application domain is power-system dispatch rather than reactor control. Applying SAC to CSTR temperature control, where manipulated variables like feed temperature, feed flow rate, coolant flow rate, or heating input must be adjusted safely and adaptively under nonlinear thermal dynamics, is motivated by the entropy-regularized SAC structure, stable actor–critic learning, and constraint-aware reward formulation. References 7–35 provide a pathway for SAC control for systems like CSTR.

^3,6,7,11,29^ highlight the growing role of RL in reactor temperature regulation, adaptive control, operational safety, and practical deployment. In ref^3^, a mechanistic-model-based Twin Delayed Deep Deterministic Policy Gradient (TD3) controller enhanced with improved Ornstein–Uhlenbeck noise, fractional-order memory, and reward-feedback adaptation improves exploration, reduces overshoot, and increases outlet-temperature tracking accuracy under cooling disturbances, although it remains dependent on accurate modeling and simulation-based validation. Reference^6^ introduces a control-informed reinforcement learning framework that embeds Proportional–Integral–Derivative (PID) structure within a deep RL policy, achieving superior set-point tracking, improved sample efficiency, reduced training variance, and enhanced robustness compared with pure model-free RL and static PID control. In ref^7^, TD3- and Soft Actor–Critic (SAC)-based adaptive PID controllers are investigated for a nonlinear Continuous Stirred Tank Heater process, where Gaussian rewards enable smoother regulation and TD3 achieves lower Integral Square Error (ISE), Integral Absolute Error (IAE), settling time, and overshoot than SAC and conventional PID, together with successful real-time validation. References 10,12,12,17 extend intelligent reactor control toward optimal tracking, constrained reliability, and computational efficiency, with ref^10^ employing Adaptive Dynamic Programming (ADP) and neural networks for constrained neuro-optimal tracking,^12,12^ developing RL-enhanced event-triggered reliability control under asymmetric input constraints, and^17^ combining multiple neural networks with RL for faster, more robust, and computationally efficient regulation than NMPC. Reference^11^ proposes a Proximal Policy Optimization-based resilient control framework for safer exothermic CSTR operation, whereas ref^29^ experimentally validates an RL-assisted NMPC strategy for a jacketed batch reactor. Collectively, refs 3,6,7,10–12,12,17,29 demonstrate that intelligent reactor control has progressed from ADP-based formulations toward temperature-focused RL, event-triggered reliability, safety-oriented resilient control, and experimentally deployable predictive control.

### 1.3 Data-Driven and Hybrid Modeling Approaches for CSTR Control

Reference^5^ presents an implicit hybrid machine learning technique for Continuous Stirred Tank Reactor (CSTR) modeling that integrates physics-based conservation laws with neural networks using an implicit trapezoidal formulation solved through automatic differentiation and Newton iterations. The proposed model captures stiff reactor dynamics more effectively than an explicit RK4-based hybrid model, producing lower training and testing prediction losses while maintaining reliable performance in the presence of noisy data. Moreover, ref^5^ demonstrates that the hybrid model can serve as a surrogate environment for Proximal Policy Optimization (PPO)-based reinforcement learning, where the learned policy performs nearly as well as plant-trained RL and surpasses real-time optimization. Thus, ref^5^ highlights the effective integration of hybrid modeling and reinforcement learning for data-efficient nonlinear reactor optimization. Reference^15^ compares one fully data-driven model and three hybrid models for catalytic *CO*_2_ methanation in a CSTR, with emphasis on prediction accuracy, training effort, and reliability. The findings in ref^15^ show that hybrid models incorporating mechanistic knowledge do not always outperform purely data-driven models, indicating that model selection should depend on the available data and the specific process-modeling problem. Reference^25^ proposes a black-box nonlinear CSTR identification method based on fractional neural networks and a newly developed fractional activation function for improved real-time modeling under challenging operating conditions. The RLF tanh-based multilayer perceptron model in ref^25^ achieves improved prediction accuracy, generalization, noise tolerance, reduced-data robustness, and faster training compared with conventional tanh and existing fractional-activation-based models, although automatic fractional-order tuning and broader closed-loop validation remain necessary. Reference^30^ introduces a Lyapunov-constrained hybrid modeling framework that combines first-principles knowledge with data-driven correction terms to learn uncertain state-dependent parameters while preserving stability and physical consistency. Using a CSTR with time-varying fouling, ref^30^ shows that the method estimates reactor trajectories and drifting heat-transfer coefficients more accurately than conventional hybrid models, thereby supporting safer temperature prediction, constraint satisfaction, and adaptive control. References 14,18,21 directly connect data-driven reactor modeling with predictive control design for nonlinear CSTR operation. In ref^14^, a neural-network-based offset-free Model Predictive Control (MPC) framework employs a Nonlinear Autoregressive Network with Exogenous Inputs (NARX) model augmented with an integrating disturbance state and a nonlinear observer to eliminate steady-state tracking offsets caused by plant–model mismatch. Simulation results in ref^14^ demonstrate superior tracking performance over nominal and baseline offset-free NARX-MPC approaches, especially when only limited-excitation training data are available. Reference^18^ develops a deep-learning-based MPC scheme using a growing deep belief network, online quadratic optimization, transfer learning, and Partial Least Squares Regression (PLSR)-based fine-tuning to improve identification, tracking, and disturbance rejection, whereas ref^21^ presents a prescribed-performance-based direct data-driven MPC method that ensures bounded tracking error and Bounded-Input Bounded-Output (BIBO) stability without requiring an explicit process model. Overall, refs 5,14,15,18,21,25,30 demonstrate that CSTR modeling has progressed from standalone black-box identification toward hybrid, stability-constrained, and control-oriented data-driven frameworks capable of supporting accurate prediction, robust temperature regulation, and advanced real-time control design.^16^

### 1.4 Experimental Validation and Practical Advancements in CSTR Monitoring and Control

Reference^7^ presents a reinforcement-learning-based adaptive PID controller for a nonlinear Continuous Stirred Tank Heater process, in which TD3 and Soft Actor–Critic (SAC) agents tune PID parameters online under varying operating conditions. The results in ref^7^ show that Gaussian rewards provide smoother temperature regulation than discrete rewards, while TD3 outperforms SAC and conventional PID control through lower Integral Square Error (ISE), Integral Absolute Error (IAE), settling time, zero overshoot in simulation, and successful real-time validation. Reference^27^ develops an experimentally validated model-based fault diagnostic framework for a closed-loop safety-critical CSTR by combining residual signal analysis with the *T* ^2^ statistic for real-time fault detection and fault-size estimation under feedback temperature control. Although ref^27^ demonstrates reliable deviation detection, accurate estimation of feed-inlet temperature faults, and effective set-point tracking under alternative manipulated-input configurations, further work is required for multivariable reactor networks, simultaneous faults, nonlinear adaptive fault-tolerant control, and industrial validation under stronger uncertainties and actuator limitations. In ref^29^, an experimentally validated reinforcement-learning-assisted nonlinear Model Predictive Control (NMPC) framework for a jacketed batch reactor employs an actor–critic algorithm and an RNN-based predictive model derived from open-loop process data to update NMPC weights dynamically. The Jetson Orin implementation in ref^29^ confirms that integrating RL with NMPC can improve computational efficiency, reduce prediction error, and enhance temperature-trajectory tracking in practical nonlinear thermal systems where accurate first-principles models are difficult to maintain. References 8,9,23 broaden the experimental foundation of reactor studies through process characterization, mixing analysis, inhibitor evaluation, and kinetic parameter estimation. Reference^8^ introduces a brine chemistry inhibitor CSTR method that improves scale-inhibitor screening through rapid crystallization, sharper end points, repeatability errors below 6.6%, approximately 20 min testing, and a mechanistic correlation for minimum inhibitor concentration prediction.^9^ experimentally investigates residence time distribution in three unequal cascade CSTRs using pulse and step tracer inputs, revealing greater nonideal deviations with pulse injection and systematic effects of flow rate on concentration and residence-time curves, whereas ref^23^ estimates reaction kinetics in an adiabatic CSTR using only transient start-up temperature measurements. Collectively, refs 7–9,23,27,29 demonstrate that experimental and practical CSTR research not only validates advanced real-time control strategies but also strengthens fault diagnosis, mixing assessment, inhibitor analysis, and thermal–kinetic characterization for reliable industrial deployment.

### 1.5 Advanced Temperature Regulation, Safety, and Reliability in CSTRs

Reference^20^ proposes an iterative-learning-observer-based predefined-time sliding mode control strategy for a Continuous Stirred Tank Reactor (CSTR) subject to input-output constraints and external disturbances, ensuring rapid temperature tracking within a designer-defined convergence time. By integrating a constraint-aware sliding surface with disturbance estimation, achieves improved response speed, tracking accuracy, and disturbance rejection over finite-time and adaptive backstepping alternatives, although robustness under model mismatch, measurement noise, and large-scale reactor configurations remains to be investigated. References 11–12 establish that reinforcement learning (RL) can improve not only control performance but also safety, reliability, and constrained reactor operation. In ref^11^, a Proximal Policy Optimization-based resilient control framework incorporates temperature, liquid-level, and thermal-runaway safety constraints into the reward design of an exothermic CSTR, enabling adaptive operation under uncertainty and outperforming PID and NMPC in stability, efficiency, and safety. References 12,12 develop RL-enhanced event-triggered reliability controllers for uncertain CSTRs with asymmetric input constraints, using nonquadratic performance functions, dynamic event-triggered Hamilton–Jacobi–Bellman formulations, and critic-only neural networks to reduce communication and execution burden while ensuring dependable constrained regulation. References 13,21,31 further extend reliable CSTR control through prescribed-performance adaptive neural-network control, direct data-driven model predictive control with bounded tracking error and BIBO stability, and gain-scheduled mean-field-type control for uncertainty-aware temperature regulation. Reference^13^ confines tracking errors within prescribed transient and steady-state limits through an invertible performance function and online radial basis function neural-network adaptation, while ref^21^ achieves accurate tracking without requiring an explicit process model. Reference^28^ addresses delayed and disturbance-sensitive reactor temperature regulation by integrating dynamic matrix control, PID cascade control, Smith prediction, and a disturbance observer, thereby reducing overshoot, shortening peak time, and improving antidisturbance performance. Reference^31^ explicitly incorporates the mean and variance of system states and inputs into a stochastic CSTR control formulation, reducing temperature fluctuations and concentration variability while supporting risk-aware closed-loop operation under disturbances. References 3,7,11,29 demonstrate the increasing importance of RL for intelligent temperature regulation in nonlinear reactor systems. In ref^3^, a mechanistic-model-based TD3 controller enhanced with improved Ornstein–Uhlenbeck noise, fractional-order memory, and reward-feedback adaptation improves outlet-temperature tracking under cooling disturbances, although its dependence on an accurate model and simulation-based validation limits immediate industrial deployment. Reference^7^ applies TD3- and SAC-based adaptive PID control to a nonlinear Continuous Stirred Tank Heater process, showing that Gaussian rewards provide smoother regulation and that TD3 achieves lower ISE, IAE, settling time, and overshoot than SAC and conventional PID, together with successful real-time validation. Reference^29^ experimentally validates an RL-assisted NMPC framework for a jacketed batch reactor, where an actor–critic algorithm dynamically updates controller weights using an RNN-based predictive model derived from open-loop reactor data. References 27,30 strengthen the temperature-control literature through real-time fault diagnosis and stable control-oriented thermal modeling, with ref^27^ employing the *T* ^2^ statistic and residual analysis for fault identification and estimation, and ref^30^ using Lyapunov-constrained hybrid modeling to improve prediction of reactor trajectories and fouling-induced heat-transfer degradation. Overall, refs 3,7,11,12,20,21,27–32 establish the CSTR as a benchmark nonlinear process whose temperature-control research has evolved from constrained model-based regulation toward resilient RL, dependable event-triggered control, data-driven predictive design, fault-aware operation, and uncertainty-conscious thermal safety management.

### 1.6 Research Gaps

Table 1 showcase the research gaps which points toward reinforcement learning based on NARX model of lab scale CSTR.

**Table 1:** Research Gaps Leading to the Proposed Work

Theme	References	Research gaps leading to the proposed work
**Model-based control**	^1,19,20,22,24,26,28,31^	Adaptive, less model-dependent CSTR temperature control is needed for uncertainties, disturbances, constraints, and practical implementation.
**RL/ADP/intelligent control**	^2–4,6,7,7–10,10,11,11,12,12,12–17,17–29,29–35^	Practical RL-based CSTR temperature control requires safe, stable, computationally efficient real-time deployment beyond simulation environments.
**Data-driven/hybrid/black-box modeling**	^5,14,15,18,21,25,30^	A simple, interpretable, control-oriented nonlinear CSTR temperature model is needed for practical closed-loop real-time implementation.
**NARX/control-oriented predictive modeling**	^14,32^	NARX enables computationally feasible, RL-based temperature control and experimental validation for lab-scale nonlinear CSTR systems.
**Experimental/practical/diagnostic studies**	^7–9,23,27,29^	Reliable real-time experimental validation of RL-based temperature control is essential for practical lab-scale CSTR deployment.
**Safety/constraints/reliability**	^11,12,20,21,31^	A safety-aware, constraint-aware adaptive RL framework is needed for reliable lab-scale CSTR temperature control operation.
**Temperature-control-focused studies**	^3,7,11,20,27–31^	RL-based temperature control with control-oriented nonlinear modeling enables safe, adaptive, real-time, reliable lab-scale CSTR operation.
**Reactor characterization/kinetics/mixing/diagnosis support**	^8,9,23,27^	Lab-scale reactor behavior insights should inform intelligent temperature control for realistic, deployable CSTR controller design.
**Integrative benchmark/summary perspective**	^32^	A unified data-driven RL framework is needed for practical real-time temperature control of lab-scale CSTRs.

The focus of this work is on developing NARX model based on the open loop data set and design of RL TD3 controller in the closed loop. Results showcases reactor temperature tracking on variation of two manipulated variables at a time. Realtime deployment is also performed. In the propoesed work NARX model is given as the environment.

The organization of the paper is as follows. Section 2 showcase the labscale CSTR setup and schematic. Section 3 highlights mathematical modeling of CSTR. Section 4 focus on development of NARX model based on the open loop data. Section 5 showcase temperature control of CSTR by RL TD3/SAC algorithm. Section 6 gives the results and discussions of temperature tracking on variation of two manipulated variables. Section 7 gives the conclusion and future scope.

## 2 CSTR Lab-Scale Pilot Plant

The CSTR considered in this work is a lab scale Double jacketed type shown in Figure 1.

**Figure 1:**
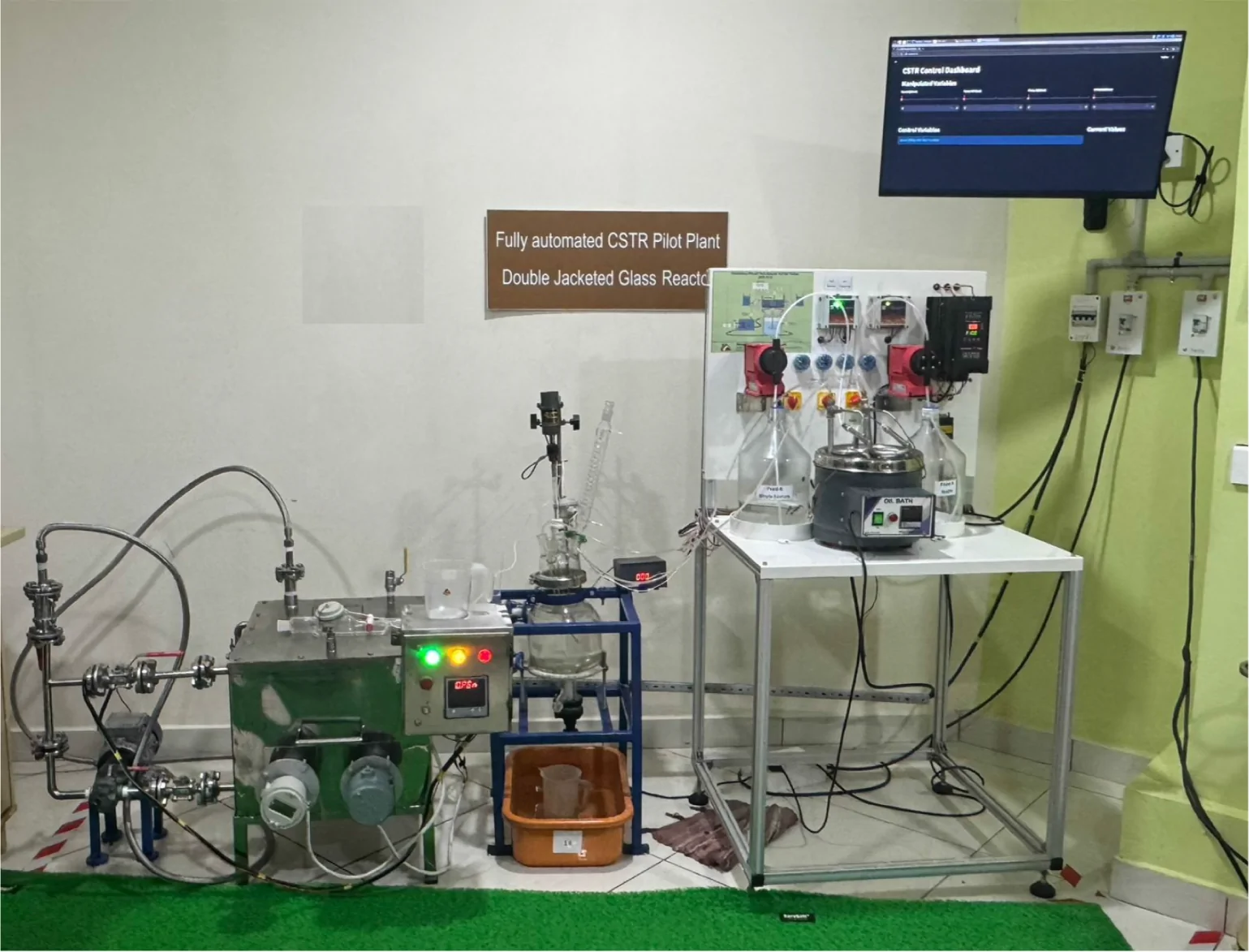
Pilot plant CSTR setup at machine learning and advanced process control lab, School of Electrical Engineering, Manipal Institute of Technology, Manipal, Karnataka. (image captured by Thirunavukkarasu Indiran).

The schematic diagram of the pilot plant CSTR is shown in Figure 2.

**Figure 2:**
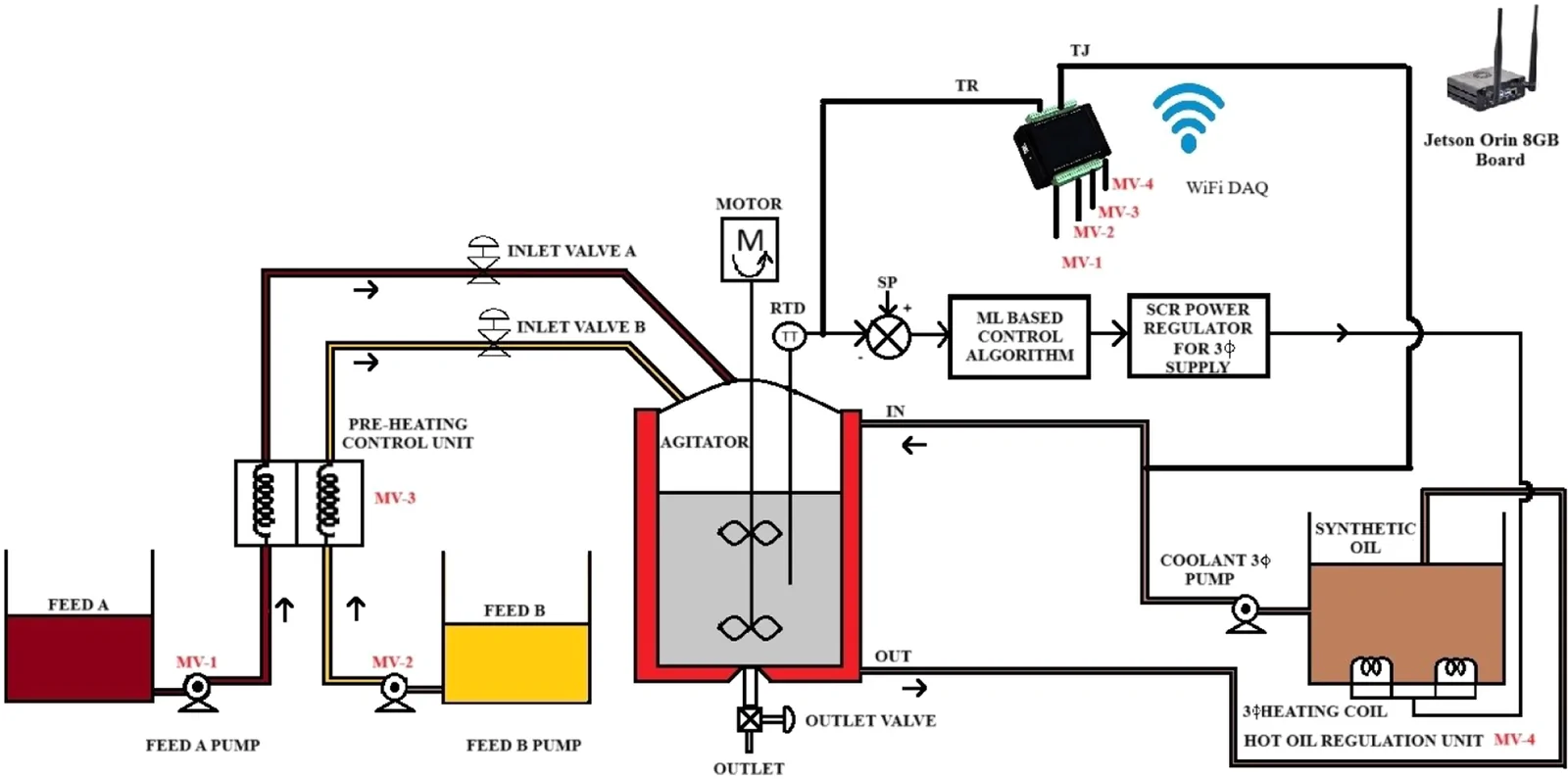
Schematic diagram of automated CSTR pilot plant with machine learning based control. (image created by Jim George).

The reactor vessel is borosilicate glass construction. This resists corrosion, which means that the vessel can handle all sorts of chemical reactions without breaking down over time. Another advantage is that the glass provides complete visibility of the process. This allows the operator to watch the reaction happening inside in real time. They can observe changes in color or any bubbles that may form. The progress of the reaction is visualized by these indicators.

The control arrangement employs a double-jacketed reactor architecture to ensure exact temperature regulation throughout operation. While the outer jacket circulates a coolant or heat-transfer fluid, such as water, oil, or another thermal medium, the inner jacket holds the reaction mixture. The system can heat or cool the reaction mixture as needed by varying the temperature of this circulating fluid. In both exothermic and endothermic processes, this promotes appropriate product production and preserves the intended reaction kinetics. Additionally, the outer jacket acts as an insulating barrier, increasing energy efficiency and lowering heat loss to the environment. Additionally, by avoiding direct exposure to extremely high or low wall temperatures, it enhances operator safety. Overall, the double-jacketed design maintains a stable, predictable, and well-controlled thermal environment within the reactor.

## 3 First-Principle Model of CSTR

The typical method to CSTR modeling is physics-based, drawing on the fundamental rules of mass and energy conservation. By applying these rules to the ideal mixed reactor system, the dynamics can be described as a set of coupled, nonlinear ordinary differential equations (ODEs).^3^ The description of variables used is given in Table 2.

**Table 2:** Description of Variables and Parameters

Symbol	Description
*V*	Reactor volume
*F*	Inlet flow rate
*C*_*A*_	Concentration of species A in the reactor
*C*_*Af*_	Feed concentration of species A
*k*_0_	Pre-exponential factor from the Arrhenius equation
*E*_*a*_	Activation energy
*R*	Universal gas constant
*T*	Reactor temperature
ρ	Fluid density in the reactor
*C*_*p*_	Specific heat of the fluid
*T*_*f*_	Inlet feed temperature
Δ*H*	Heat of reaction
*Q*	Heat removed by the cooling jacket

a) Expression for Mass Balance: the mass balance accounts for the buildup of reactant species in the reactor, which is caused by intake flow, output flow, and reactant consumption as a result of the chemical reaction. It is often phrased as in eq 1.

1 $$V\frac{dC_{A}}{dt}=F\left(C_{Af}-C_{A}\right)-Vk_{0}e^{-E_{a}/RT}C_{A}$$

b) Expression for Energy Balance: the energy balance determines thermal dynamics. It accounts for the heat brought in by the feed, the heat removed by the product stream, the heat produced by the exothermic process, and the heat removed by the cooling jacket. The expression is given by eq 2.

2 $$V\rho C_{p}\frac{dT}{dt}=F\rho C_{p}\left(T_{f}-T\right)+\left(-\Delta H\right)Vk_{0}e^{-E_{a}/RT}C_{A}-Q$$

While these ODEs provide a rigid theoretical foundation, unmodeled dynamics and parameter variations often require the transition to empirical, data-driven modeling paradigms.

## 4 Nonlinear Autoregressive Exogenous Model of CSTR

NARX modeling results for the reactor temperature time series *T*_*r*_(*k*) of a lab scale Continuous Stirred Tank Reactor (CSTR) shown in Figure 1, using multivariate time-series inputs is performed based on ref^32^. The objectives are (i) develop a high-accuracy NARX model for open-loop prediction of *T*_*r*_(*k*); (ii) derive a nonlinear differential equation approximation using a second NARX model trained on Δ*T*_*r*_(*k*) = *T*_*r*_(*k*+1)-*T*_*r*_(*k*); (iii) report all estimated parameters (including all rows and columns of matrices) without omission; (iv) interpret all training/validation figures and provide the algorithmic workflow used to develop the NARX model.

### 4.1 Time-Series Inputs and NARX Formulation

The exogenous time-series input vector used in the NARX model is

3 $$u\left(k\right)={\left[T_{j}(k)\;T_{B,in}(k)\;T_{A,in}(k)\;u_{HOB}(k)\;u_{H}(k)\;F_{A}(k)\;F_{B}(k)\;\right]}^{T}$$

The discrete-time NARX model for reactor temperature is

4 $${\mathrm{T̂}}_{r}\left(k\right)=F\left(T_{r}\left(k-1\right),...,T_{r}\left(k-n_{y}\right),\;u\left(k-1\right),...,u\left(k-n_{u}\right)\right)$$

where F(·) is implemented as a neural network (hidden layer nonlinearity, output layer linear **purelin**). With delays:

$$D_{u}=\left\{1,2,...,15\right\},D_{y}=\left\{1,2,...,15\right\}$$

the regressor vector is formed by stacking delayed samples:

5 $$\phi \left(k\right)=\left[\begin{array}{l} u\left(k-d\right),d\in D_{u}\\ T_{r}\left(k-d\right),d\in D_{y} \end{array}\right]\in R^{|\phi |}$$

### 4.2 Algorithm: Developing the NARX Model

Algorithm 1 describes the complete workflow used to build the NARX model for *T*_*r*_(*k*) from the time-series data set..
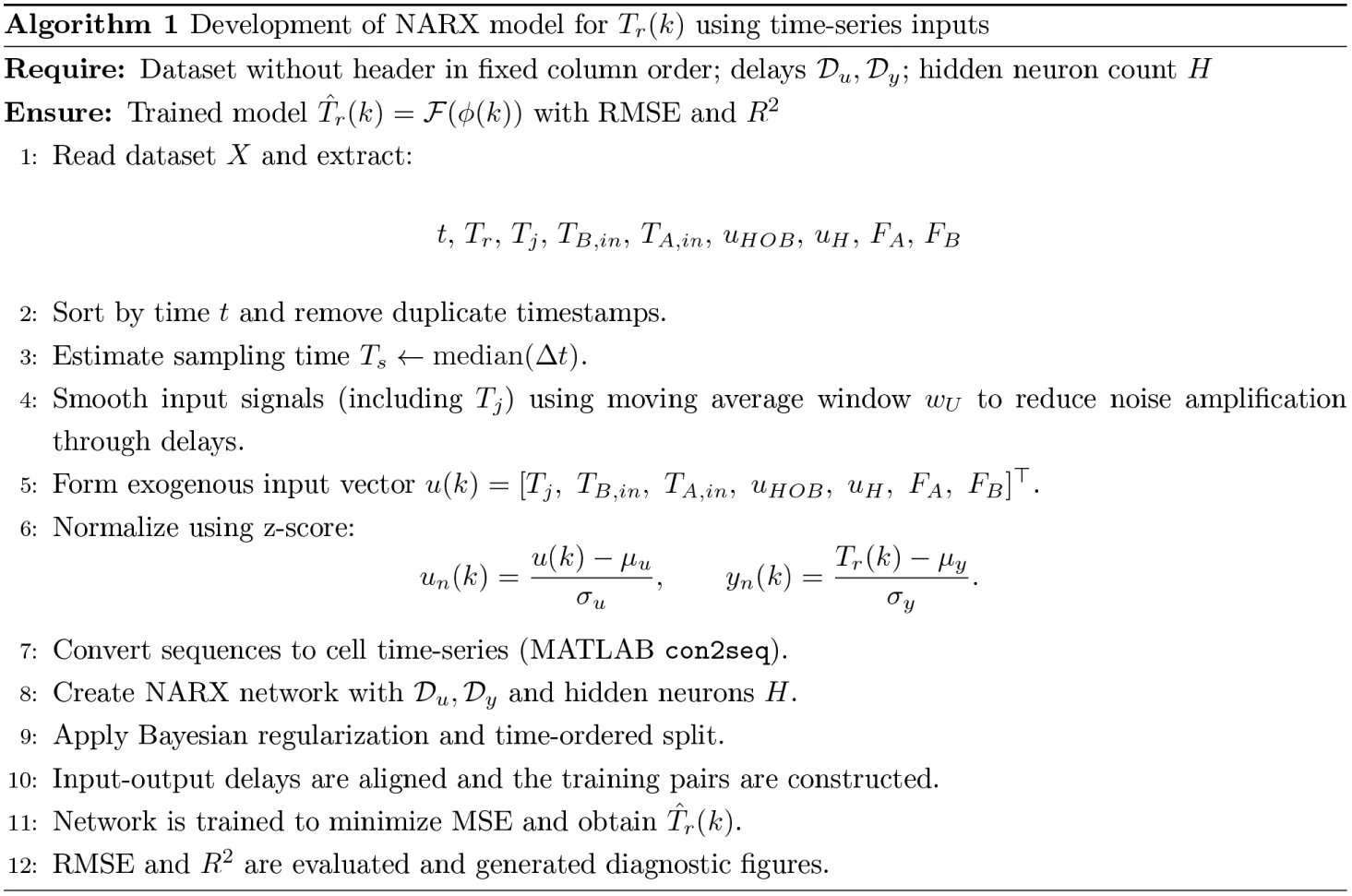


### 4.3 NARX-Derived Nonlinear Differential Equation

A second NARX model is trained to predict:

$$\Delta T_{r}\left(k\right)=T_{r}\left(k+1\right)-T_{r}\left(k\right)$$

Using the sampling time *T*_*s*_, the nonlinear differential equation approximation is

6 $$\frac{dT_{r}}{dt}{|}_{t=t_{k}}\approx \frac{1}{T_{s}}\Delta {\mathrm{T̂}}_{r}\left(k\right)$$

with the NARX network output for Δ*T*_*r*_ expressed as

7 $$\Delta {\mathrm{T̂}}_{r}\left(k\right)=\mu_{D}+\sigma_{D}\left(W_{2D}\left(W_{1D}\phi \left(k\right)+b_{1D}\right)+b_{2D}\right)$$

where ϕ­(*k*) stacks the delayed time-series regressors (delayed *u* and delayed Δ*T*_*r*_ according to MATLAB internal ordering created by preparets).

### 4.4 Full Parameter Values

Values can be found in Table 3.

**Table 3:** Scalar Parameters

Parameter	Value
*T*_*s*_	1.00
μ_*D*_	0.005748098056
σ_*D*_	0.041486521948
*b*_2*D*_	0.114187139156
*H*	20
|ϕ(*k*)|	90

### 4.5 Differential Equation

Since *T*_*s*_ = 1, eq 6 becomes

8 $$\frac{dT_{r}}{dt}{|}_{t=t_{k}}=\Delta {\mathrm{T̂}}_{r}\left(k\right)$$

Substituting the scalar values into eq 7:

9 $$\Delta {\mathrm{T̂}}_{r}\left(k\right)=0.005748098056+0.041486521948\left(W_{2D}\left(W_{1D}\phi \left(k\right)+b_{1D}\right)+0.114187139156\right)$$

where *W*_2*D*_, *b*_1*D*_ and the full *W*_1*D*_ matrix are listed in Tables 4, 5 and Table 15 respectively. The open-loop profile (Figure 3) demonstrates that the NARX model output overlaps the measured reactor temperature throughout the experiment. The nearly identical curves indicate that the selected time-delay embedding and the nonlinear mapping in eq 4 capture the dominant CSTR thermal dynamics. This is consistent with the quantitative performance RMSE = 0.029098 and *R* ^2^ = 0.999972, which indicate extremely low error relative to the full operating range (approximately 28°C to 46 °C). The performance curve (Figure 4) shows the evolution of mean squared error over training epochs. The reported best training performance 2.6414 × 10^–5^ at epoch 428 implies that the optimization converged and achieved a very low error. The separation between training and test curves provides a visual indication of generalization. Because the test curve remains low and stable after convergence (instead of diverging), the trained model does not exhibit severe overfitting. Bayesian regularization helps to smooth out weight distributions and reduces noise sensitivity.

**Table 4:** Output Layer Weights *W*_2*D*_ (1 × 20)

Index	*W*_2*D*_	Index	*W*_2*D*_
1	–0.7253	11	0.7568
2	–0.5301	12	0.7231
3	0.5867	13	0.7265
4	–0.7760	14	–0.5567
5	–0.7483	15	–0.6457
6	–0.8383	16	–0.6361
7	–0.8337	17	0.4965
8	0.5234	18	–0.7248
9	0.7890	19	–0.7348
10	0.7296	20	0.8244

**Table 5:** Hidden Bias Vector *b*_1*D*_ (20 × 1)

Index	*b*_1*D*_	Index	*b*_1*D*_
1	0.0523	11	0.0651
2	0.0060	12	0.0918
3	–0.2398	13	0.1351
4	–0.0720	14	0.0212
5	–0.1384	15	–0.0541
6	0.0669	16	–0.1005
7	–0.1059	17	0.0239
8	0.0856	18	0.0122
9	0.0486	19	0.1944
10	–0.0333	20	–0.1058

**Figure 3:**
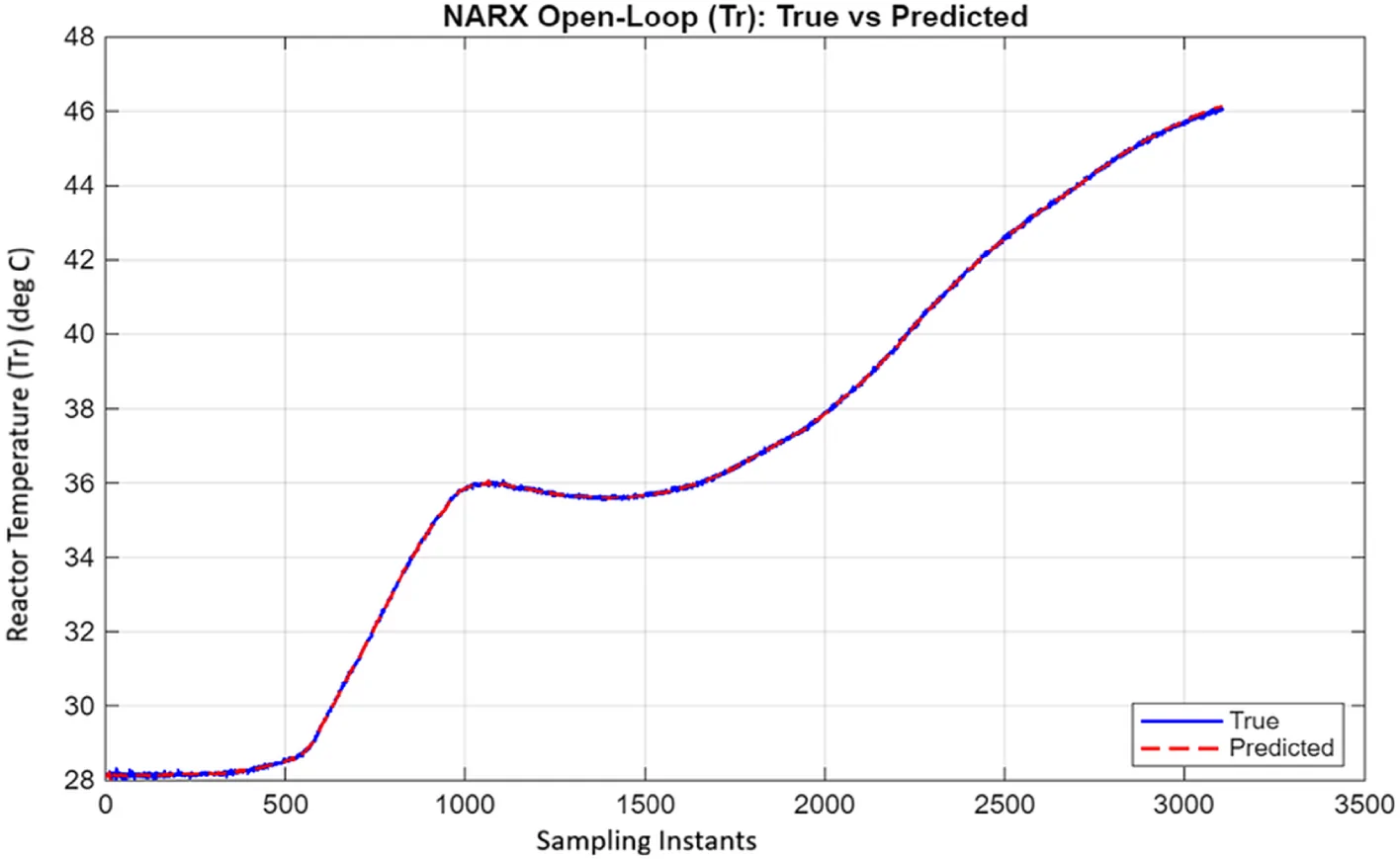
NARX open-loop profile of reactor temperature: measured *T_r_* (*k*) vs predicted *T̂_r_* (*k*) after delay alignment. Near-perfect overlap matches RMSE = 0.029098 and *R* ^2^ = 0.999972.

**Figure 4:**
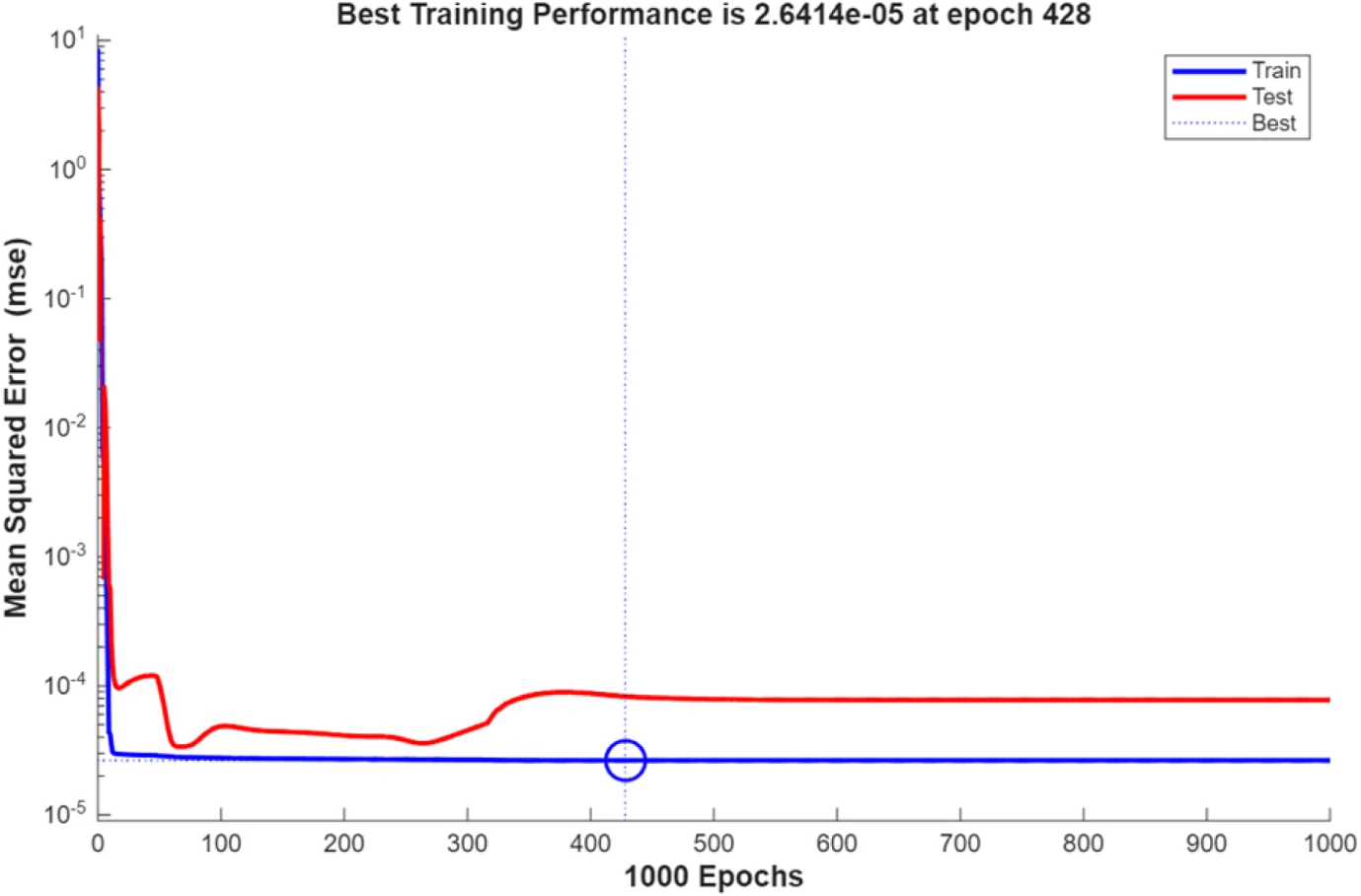
Training performance curve (MSE vs epochs). Best training performance is 2.6414 × 10^–5^ at epoch 428. Stable low test error indicates good generalization under time-ordered split.

The error histogram (Figure 5) depicts the distribution of residuals. A narrow distribution centered at zero shows little bias and consistent prediction accuracy. The overlap of training and test residual distributions shows similar error statistics for visible and unseen segments, indicating that the model is reliable for prediction. The regression graphs (Figure 6) compare expected outputs against objectives for training, test, and all data. The strong correlation coefficients (*R* near to 1) show that the anticipated values are extremely close to the ideal line *Y* = *T*. Small variations in the test regression line from the ideal line may occur as a result of time-ordered splitting and regime shifts, but the plot demonstrates the general high agreement.

**Figure 5:**
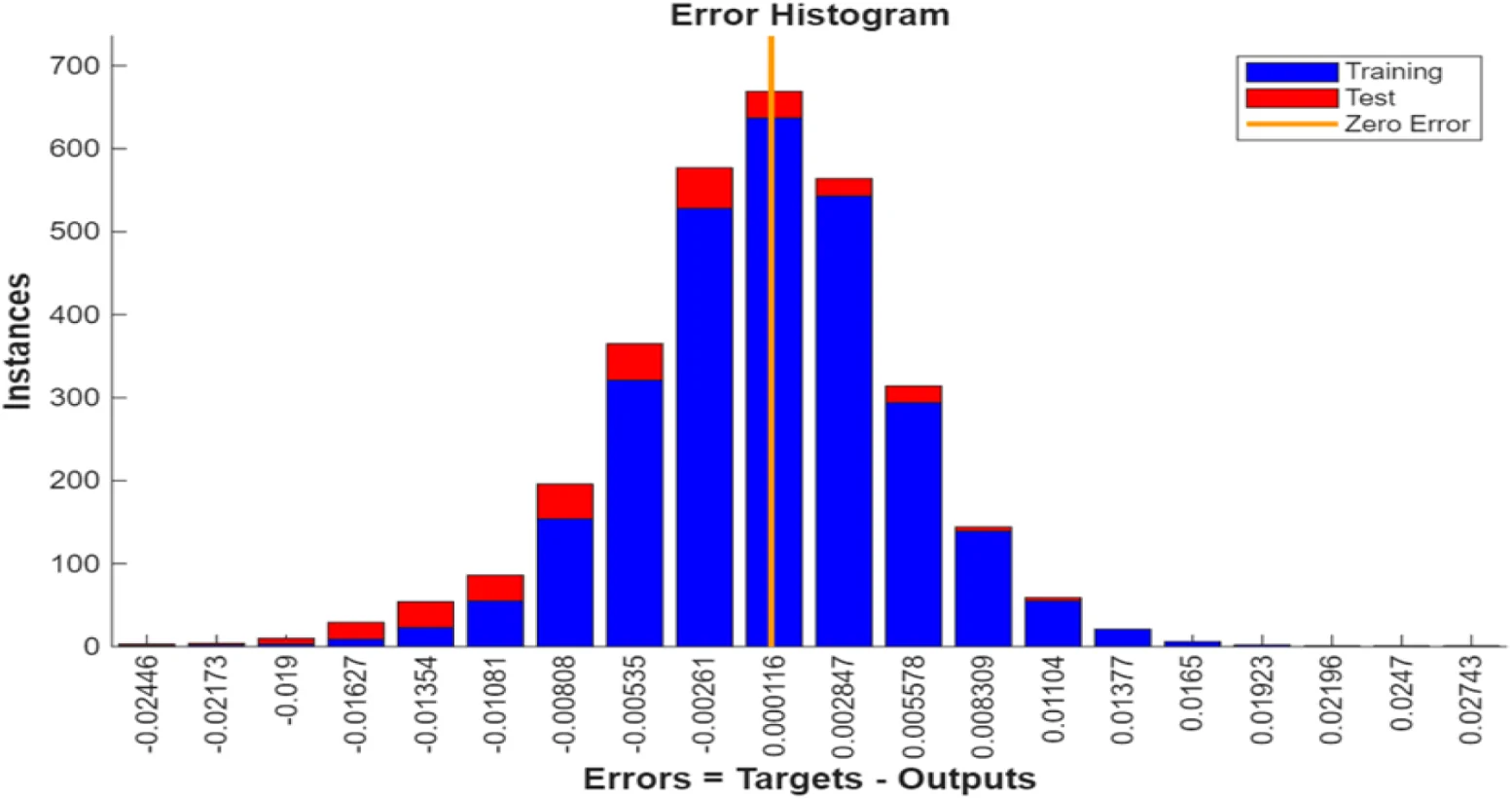
Error histogram for training and test sets. Errors concentrated near zero indicate negligible bias and high consistency of predictions.

**Figure 6:**
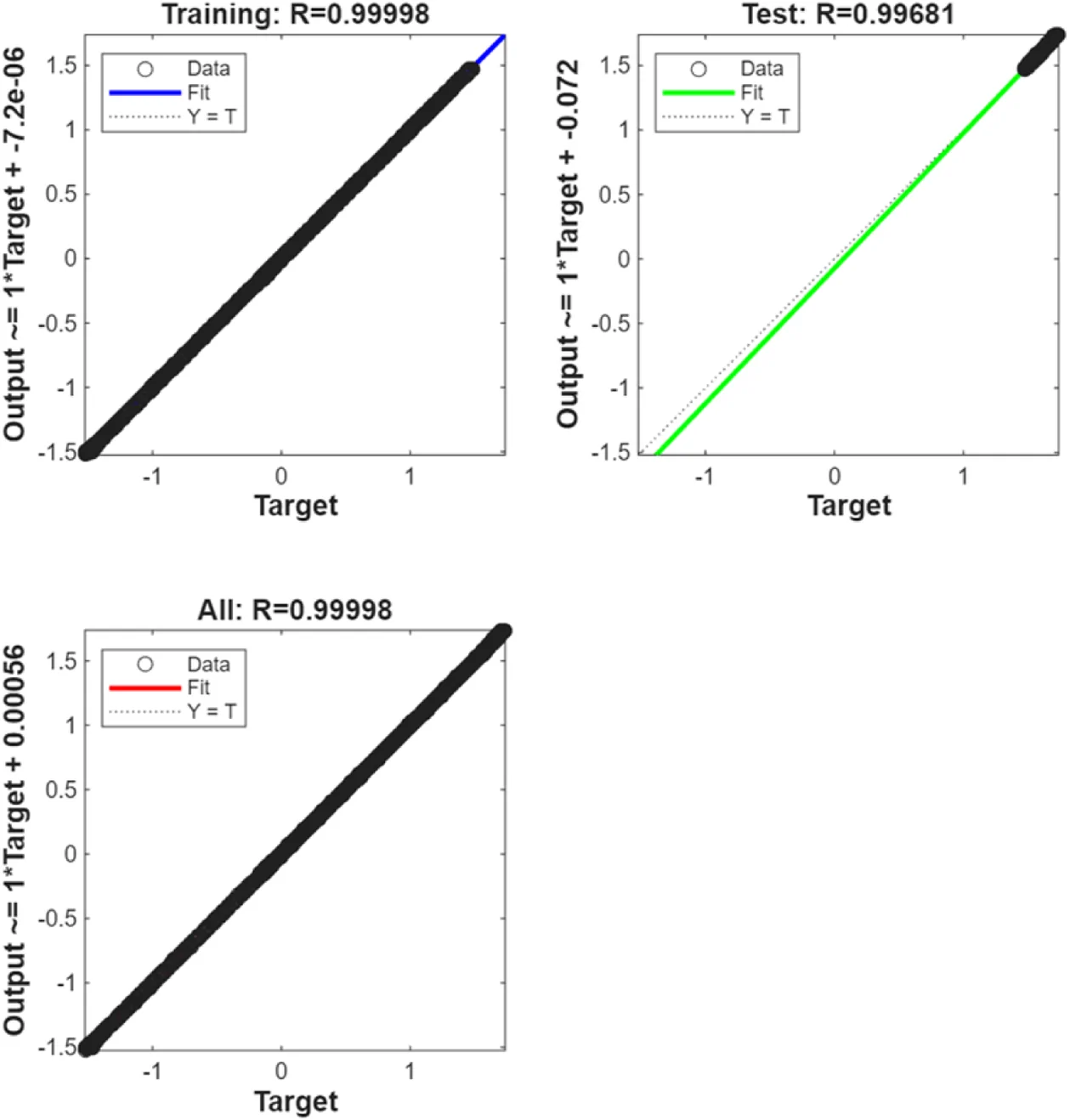
Regression plots (output vs target) for training, test, and all samples. High correlation indicates strong agreement between predicted and measured reactor temperature.

Finally, the network architecture diagram (Figure 7) demonstrates the structural meaning of the NARX model: delayed time-series inputs and delayed output feedback are weighted, biased, combined, passed through a nonlinear hidden layer (20 neurons), and then mapped by a linear output layer to produce the single output *T*_*r*_. This is closely related to eq 4 and gives a clear conceptual explanation of how time-series memory is integrated in the predictor (Table 6). Table 6 presents the performance of NARX and Knowledged based model.

**Table 6:** Performance Metrics of the NARX Model and Derived ODE

Quantity	RMSE	*R* ^2^
Sampling Time, *T*_*s*_	1.00	
*T*_*r*_(*k*)	0.029098	0.999972
Δ*T*_*r*_(*k*)	0.028383	0.527426
$\frac{dT_{r}}{dt}$	0.028383	0.527426

**Figure 7:**
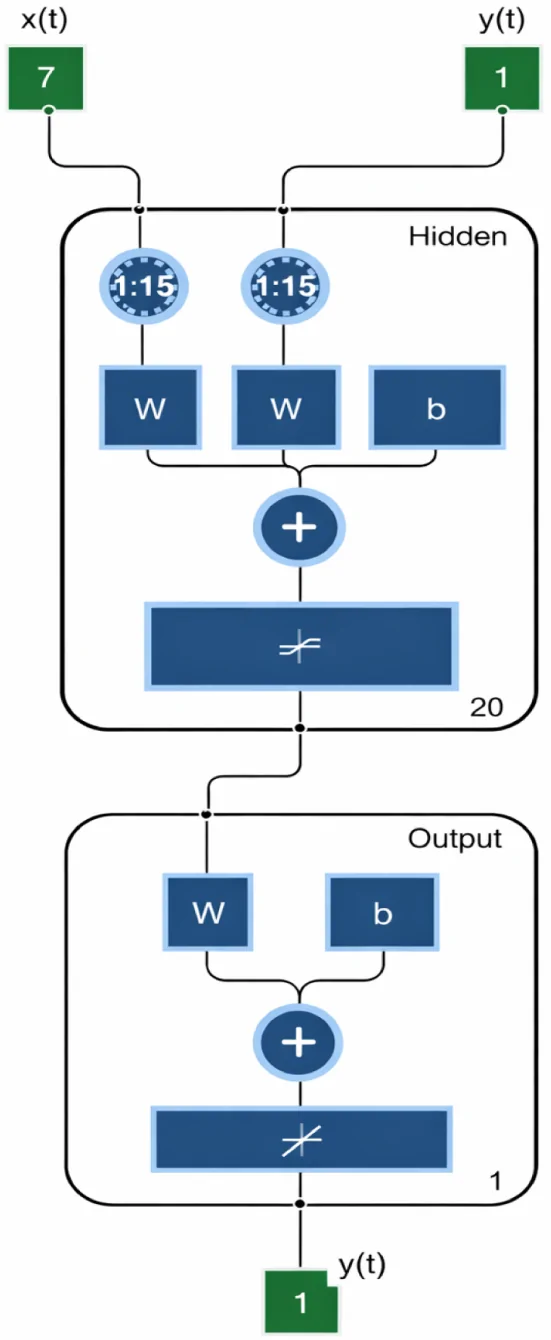
NARX network architecture. Exogenous input time series (*x*(*t*), 7 channels) and output feedback (*y*(*t*), 1 channel) pass through delays, are weighted and biased, processed by a 20-neuron nonlinear hidden layer, and mapped to a single linear output *T̂_r_*.

A high-fidelity NARX model was obtained for reactor temperature prediction with RMSE = 0.029098 and *R* ^2^ = 0.999972, demonstrating near-perfect open-loop tracking from time-series inputs. A NARX-based nonlinear differential equation approximation was also formed by modeling Δ*T*_*r*_(*k*) and converting it to *dT*_*r*_/*dt* via *T*_*s*_. The complete parameter set defining the differential equation approximation is reported without omission, including the full $W_{1D}\in R^{20\times 90}$ matrix, the bias vector *b*_1*D*_, and the output weights *W*_2*D*_.

## 5 Temperature Control of CSTR by Reinforcement Learning

The trained NARX model of the CSTR is the reinforcement learning environment. The goal is to make the reactor temperature follow a time-varying reference trajectory while maintaining the manipulated variables within experimentally valid operating limits. Two manipulated variables are chosen at a time.

### 5.1 Control Problem Formulation

The reactor temperature set point is defined as

10 Tsp(k)={30C°,0≤k<60,35C°,60≤k<120,43C°,120≤k<180,37C°,180≤k<240,40C°,240≤k≤360.

The controlled variable is the reactor temperature:

11 $$y\left(k\right)=T_{r}\left(k\right)$$

The reference signal is

12 $$r\left(k\right)=T_{sp}\left(k\right)$$

The manipulated-variable vector is defined as

13 $$u\left(k\right)=\left[\begin{array}{l} u_{1}\left(k\right)\\ u_{2}\left(k\right) \end{array}\right]$$

The pair (*u*_1_,*u*_2_) is selected from the experimentally available manipulated-variable combinations:

14 $$\left(u_{1},u_{2}\right)\in \{\begin{array}{l} \left(\mathrm{Feed A Temperature},\mathrm{Feed B Temperature}\right)\\ \left(\mathrm{Feed A Temperature},\mathrm{Feed B Flow rate}\right)\\ \left(\mathrm{Feed B Temperature},\mathrm{Feed A Flow rate}\right)\\ \left(\mathrm{Feed A Flow rate},\mathrm{Feed B Flow rate}\right)\\ \left(\mathrm{Hot Oil Bath},\mathrm{Feed A Temperature}\right)\\ \left(\mathrm{Hot Oil Bath},\mathrm{Feed B Temperature}\right)\\ \left(\mathrm{Hot Oil Bath},\mathrm{Feed A Flow rate}\right)\\ \left(\mathrm{Hot Oil Bath},\mathrm{Feed B Flow rate}\right) \end{array}$$

The reactor-temperature tracking error is

15 $$e\left(k\right)=T_{sp}\left(k\right)-T_{r}\left(k\right)$$

The desired closed-loop tracking condition is

16 $$T_{r}\left(k\right)\approx T_{sp}\left(k\right),\;k=1,2,...,N$$

The reinforcement learning agent generates the normalized action vector

17 $$a\left(k\right)=\left[\begin{array}{l} a_{1}\left(k\right)\\ a_{2}\left(k\right) \end{array}\right],{\;a}_{i}\left(k\right)\in \left[0,100\right],\;i=1,2$$

The normalized action is mapped into the real manipulated-variable range as

18 $$u_{i}^{cmd}\left(k\right)=u_{i,\min}+\frac{a_{i}\left(k\right)}{100}\left(u_{i,\max}-u_{i,\min}\right),\;i=1,2$$

To avoid abrupt actuator movement, first-order smoothing is applied:

19 $$u_{i}\left(k\right)=0.5u_{i}\left(k-1\right)+0.5u_{i}^{cmd}\left(k\right),\;i=1,2$$

The smoothed manipulated variable is saturated as

20 $$u_{i}\left(k\right)=\min\left[\max\left(u_{i}\left(k\right),u_{i,\min}\right),{\;u}_{i,\max}\right]$$

Thus,

21 $$u_{i,\min}\leq u_{i}\left(k\right)\leq u_{i,\max},\;i=1,2$$

The NARX regressor vector is represented as

22 $$\phi \left(k\right)\in R^{90}$$

At each sampling instant, the current manipulated variables and reactor temperature are inserted into the regressor:

23 $$\phi \left(k\right)={[...,u_{1}(k),u_{2}(k),T_{r}(k)]}^{T}$$

The reinforcement learning state is the normalized NARX regressor:

24 $$s\left(k\right)=\frac{\phi \left(k\right)}{50},\;s\left(k\right)\in R^{90}$$

The hidden-layer output of the trained NARX model is

25 $$h\left(k\right)=\tanh\left(W_{1D}\phi \left(k\right)+b_{1D}\right)$$

The predicted reactor-temperature increment is

26 $$\Delta T_{r}\left(k\right)=\mu_{D}+\sigma_{D}\left(W_{2D}^{T}h\left(k\right)+b_{2D}\right)$$

The NARX prediction can be compactly represented as

27 $$T_{r}\left(k+1\right)=f_{\mathrm{NARX}}\left(\phi \left(k\right),u_{1}\left(k\right),u_{2}\left(k\right)\right)$$

The integral and derivative tracking-error terms are

28a $$I_{e}\left(k\right)=I_{e}\left(k-1\right)+e\left(k\right)$$

28b Δe(k)=e(k)−e(k−1)

The auxiliary stabilizing signal is

29 $$v_{PID}\left(k\right)=K_{p}e\left(k\right)+K_{i}I_{e}\left(k\right)+K_{d}\Delta e\left(k\right)$$

The midpoint value of each manipulated variable is

30 $$u_{i,\mathrm{mid}}=\frac{u_{i,\min}+u_{i,\max}}{2},\;i=1,2$$

The normalized manipulated-variable contribution is

31 $$v_{u}\left(k\right)=0.05\left[\frac{u_{1}\left(k\right)-u_{1,\mathrm{mid}}}{u_{1,\max}-u_{1,\min}}+\frac{u_{2}\left(k\right)-u_{2,\mathrm{mid}}}{u_{2,\max}-u_{2,\min}}\right]$$

The closed-loop NARX environment update is

32 $$T_{r}\left(k+1\right)=T_{r}\left(k\right)+\Delta T_{r}\left(k\right)+v_{u}\left(k\right)+0.7v_{PID}\left(k\right)-0.07T_{r}\left(k\right)$$

The manipulated-variable penalty is

33 $$J_{u}\left(k\right)={\left(\frac{u_{1}\left(k\right)-u_{1,\mathrm{mid}}}{u_{1,\max}-u_{1,\min}}\right)}^{2}+{\left(\frac{u_{2}\left(k\right)-u_{2,\mathrm{mid}}}{u_{2,\max}-u_{2,\min}}\right)}^{2}$$

The reward function is

34 $$R\left(k\right)=-5e^{2}\left(k\right)-0.5J_{u}\left(k\right)$$

The reinforcement learning objective is to maximize the expected discounted cumulative reward:

35 $$J\left(\pi \right)=E_{\pi }\left[\overset{N-1}{\underset{k=0}{\sum }}\gamma^{k}R\left(k\right)\right]$$

Equivalently, the problem can be written as

36 $$\underset{\pi }{\min}E_{\pi }\left[\overset{N-1}{\underset{k=0}{\sum }}\gamma^{k}\left(5e^{2}\left(k\right)+0.5J_{u}\left(k\right)\right)\right]$$

The complete optimal control problem is

37 $$\begin{array}{ll} & \pi^{*}=\arg\underset{\pi }{\max}E_{\pi }\left[\overset{N-1}{\underset{k=0}{\sum }}\gamma^{k}R\left(k\right)\right]\\ \mathrm{subject to} & T_{r}\left(k+1\right)=f_{\mathrm{NARX}}\left(\phi \left(k\right),u_{1}\left(k\right),u_{2}\left(k\right)\right),\\ & e\left(k\right)=T_{sp}\left(k\right)-T_{r}\left(k\right),\\ & u_{i}^{cmd}\left(k\right)=u_{i,\min}+\frac{a_{i}\left(k\right)}{100}\left(u_{i,\max}-u_{i,\min}\right),\\ & u_{i,\min}\leq u_{i}\left(k\right)\leq u_{i,\max},\;i=1,2,\\ & a_{i}\left(k\right)\in \left[0,100\right],\;i=1,2 \end{array}$$

For SAC, the stochastic policy is

38 $$a\left(k\right)∼\pi_{\theta }\left(a|s\left(k\right)\right)$$

The SAC objective is

39 $$\pi^{*}=\arg\underset{\pi }{\max}E_{\pi }\left[\overset{N-1}{\underset{k=0}{\sum }}\gamma^{k}\left(R\left(k\right)+\alpha H\left(\pi \left(\cdot |s\left(k\right)\right)\right)\right)\right]$$

For TD3, the deterministic policy is

40 $$a\left(k\right)=\mu_{\theta }\left(s\left(k\right)\right)$$

The TD3 objective is

41 $$\mu^{*}=\arg\underset{\mu }{\max}E_{\mu }\left[\overset{N-1}{\underset{k=0}{\sum }}\gamma^{k}R\left(k\right)\right]$$

Thus, the final closed-loop control objective is

42 $$\mathrm{Find an RL policy that generates}\;u_{1}\left(k\right),u_{2}\left(k\right)\;\mathrm{such that}\;T_{r}\left(k\right)\to T_{sp}\left(k\right)$$

### 5.2 SAC/TD3 Algorithm

For both SAC and TD3, the same manipulated-variable mapping, reward function, NARX environment, and control problem formulation are employed. As a result, only the learning-update and policy-generation components are divided.

**Step 1: Initialize the NARX model and control problem.** Use the trained NARX parameters in refs ([Disp-formula eq25])–([Disp-formula eq26]), the set point in ref ([Disp-formula eq10]), the selected MV pair in ref ([Disp-formula eq14]), and the MV limits in ref ([Disp-formula eq21]).

**Step 2: Select the reinforcement learning controller.**

43 $$A_{RL}\in \left\{\mathrm{SAC},\mathrm{TD}3\right\}$$

**Step 3: Initialize the replay buffer.**

44 D=ϕ

**Step 4: Initialize the actor and critic networks.**

For SAC:

45 $$\pi_{\theta }\left(a|s\right),{\;Q}_{\psi_{1}}\left(s,a\right),{\;Q}_{\psi_{2}}\left(s,a\right)$$

For TD3:

46 $$\mu_{\theta }\left(s\right),\;Q_{\psi_{1}}\left(s,a\right),\;Q_{\psi_{2}}\left(s,a\right)$$

**Step 5: Initialize target networks.**

47 $${\mathrm{\psi ̅}}_{1}\leftarrow \psi_{1},\;{\mathrm{\psi ̅}}_{2}\leftarrow \psi_{2}$$

For TD3:

48 θ̅←θ

**Step 6: Set common and algorithm-specific hyperparameters.**

49 γ=0.995,τ=0.005

For SAC:

50 α=0.2

For TD3:

51 $$d=2,{\;\sigma }_{\epsilon }=2.0,\;c=5.0$$

**Step 7: Reset the environment at the beginning of each episode.**

52 Tr(0)=28⁡C°,⁣ϕ(0)=0,⁣Ie(0)=0,⁣e(−1)=0

**Step 8: Compute the current RL state.** Use the state definition in ref ([Disp-formula eq24]):

53 $$s\left(k\right)=\frac{\phi \left(k\right)}{50}$$

**Step 9: Generate the normalized action.**

For SAC:

54 ϵ∼N(0,I)

55 $$z\left(k\right)=\mu_{\theta }\left(s\left(k\right)\right)+\sigma_{\theta }\left(s\left(k\right)\right)⊙\epsilon$$

56 a(k)=50tanh(z(k))+50

For TD3:

57 $$a_{raw}\left(k\right)=50\tanh\;\left(\mu_{\theta }\left(s\left(k\right)\right)\right)+50$$

58 $$a\left(k\right)=a_{raw}\left(k\right)+\eta \left(k\right),\;\eta \left(k\right)∼N\left(0,\sigma_{\eta }^{2}I\right)$$

The action is saturated using ref ([Disp-formula eq17]):

59 $$a\left(k\right)={\mathrm{sat}}_{[0,100]}\;(a\left(k\right))$$

Step 10: Map the action to the actual manipulated variables. Use refs ([Disp-formula eq18]), ([Disp-formula eq19]), and ([Disp-formula eq20]) to obtain *u*_1_(*k*) and *u*_2_(*k*).

Step 11: Evaluate tracking error and update the NARX regressor. Use refs ([Disp-formula eq15]), (^28^), and ([Disp-formula eq23]).

**Step 12: Predict and update the reactor temperature.** Use the NARX eqs ([Disp-formula eq25])–([Disp-formula eq26]) and the closed-loop update eq ([Disp-formula eq33]).

**Step 13: Compute the reward.** Use the MV penalty in ref ([Disp-formula eq34]) and the reward in ref ([Disp-formula eq35]).

**Step 14: Store the transition in the replay buffer.**

60 $$D\leftarrow D\cup \left\{s\left(k\right),\;a\left(k\right),\;R\left(k\right),\;s\left(k+1\right),{\;d}_{k}\right\}$$

**Step 15: Sample a mini-batch from the replay buffer.**

61 $${\{s_{j},a_{j},R_{j},s_{j}^{\prime },d_{j}\}}_{j=1}^{B}∼D$$

**Step 16: Apply SAC update if SAC is selected.**

Sample the next action:

62 $$a_{j}^{\prime }∼\pi_{\theta }\left(\cdot |s_{j}^{\prime }\right)$$

Compute the SAC target:

63 $$y_{j}^{SAC}=R_{j}+\gamma \left(1-d_{j}\right)\left[\underset{i=1,2}{\min}Q_{{\mathrm{\psi ̅}}_{i}}\left(s_{j}^{\prime },a_{j}^{\prime }\right)-\alpha \log\pi_{\theta }\left(a_{j}^{\prime }|s_{j}^{\prime }\right)\right]$$

Update the SAC critic:

64 $$L_{Q}^{SAC}\left(\psi_{i}\right)=\frac{1}{B}\overset{B}{\underset{j=1}{\sum }}{[Q_{\psi_{i}}(s_{j},a_{j})-y_{j}^{SAC}]}^{2},\;i=1,2$$

Update the SAC actor:

65 $$L_{\pi }\left(\theta \right)=\frac{1}{B}\overset{B}{\underset{j=1}{\sum }}\left[\alpha \log\pi_{\theta }\left(a_{j}|s_{j}\right)-\underset{i=1,2}{\min}Q_{\psi_{i}}\left(s_{j},a_{j}\right)\right]$$

**Step 17: Apply TD3 update if TD3 is selected.**

Generate the target action:

66 $$a_{j}^{\prime }=50\tanh\left(\mu_{\mathrm{\theta ̅}}\left(s_{j}^{\prime }\right)\right)+50$$

Apply target-policy smoothing:

67 $$\epsilon_{j}∼N\left(0,\sigma_{\epsilon }^{2}I\right),\;{\overset{∼}{\epsilon }}_{j}=\mathrm{clip}\left(\epsilon_{j},-c,c\right)$$

68 $${ã}_{j}^{\prime }={\mathrm{sat}}_{[0,100]}\left(a_{j}^{\prime }+{\overset{∼}{\epsilon }}_{j}\right)$$

Compute the TD3 target:

69 $$y_{j}^{TD3}=R_{j}+\gamma \left(1-d_{j}\right)\underset{i=1,2}{\min}Q_{{\mathrm{\psi ̅}}_{i}}\left(s_{j}^{\prime },{ã}_{j}^{\prime }\right)$$

Update the TD3 critic:

70 $$L_{Q}^{TD3}\left(\psi_{i}\right)=\frac{1}{B}\overset{B}{\underset{j=1}{\sum }}{[Q_{\psi_{i}}(s_{j},a_{j})-y_{j}^{TD3}]}^{2},\;i=1,2$$

Apply delayed actor update:

71 mod(l,d)=0

When ref ([Disp-formula eq72]) is satisfied, update the actor:

72 $$L_{\mu }\left(\theta \right)=-\frac{1}{B}\overset{B}{\underset{j=1}{\sum }}Q_{\psi_{1}}\left(s_{j},\mu_{\theta }\left(s_{j}\right)\right)$$

**Step 18: Soft-update the target networks.** For SAC and TD3 critics:

73 $${\mathrm{\psi ̅}}_{i}\leftarrow \tau \psi_{i}+\left(1-\tau \right){\mathrm{\psi ̅}}_{i},\;i=1,2$$

For TD3 actor:

74 θ̅←τθ+(1−τ)θ̅

**Step 19: Repeat the training process.**

75 $$k=0,1,...,T_{s}-1,\;ep=1,2,...,N_{ep}$$

**Step 20: Perform deterministic evaluation after training.**

For SAC:

76 $$a_{SAC}\left(k\right)=50\tanh\;(\mu_{\theta }\left(s\left(k\right)\right))+50$$

For TD3:

77 $$a_{TD3}\left(k\right)=50\tanh\left(\mu_{\theta }\left(s\left(k\right)\right)\right)+50$$

**Step 21: Record the closed-loop outputs and performance variables.**

78 $$T_{r}\left(k\right),\;T_{sp}\left(k\right),\;e\left(k\right),R\left(k\right),\;u_{1}\left(k\right),\;u_{2}\left(k\right)$$

## 6 Results and Discussions

### 6.1 Manipulated Variables—Feed A Temperature, Feed B Temperature

The closed-loop performance of the SAC and TD3 controllers for CSTR reactor temperature tracking is compared in Figure 8 utilizing Feed A and Feed B temperatures as controlled variables. The stepwise reactor temperature set point can be effectively tracked by both controls. Both reinforcement learning techniques can successfully control the nonlinear CSTR temperature, as evidenced by the reactor temperature after the reference changes at roughly 30 °C, 35 °C, 43 °C, 37 °C, and 40 °C.

**Figure 8:**
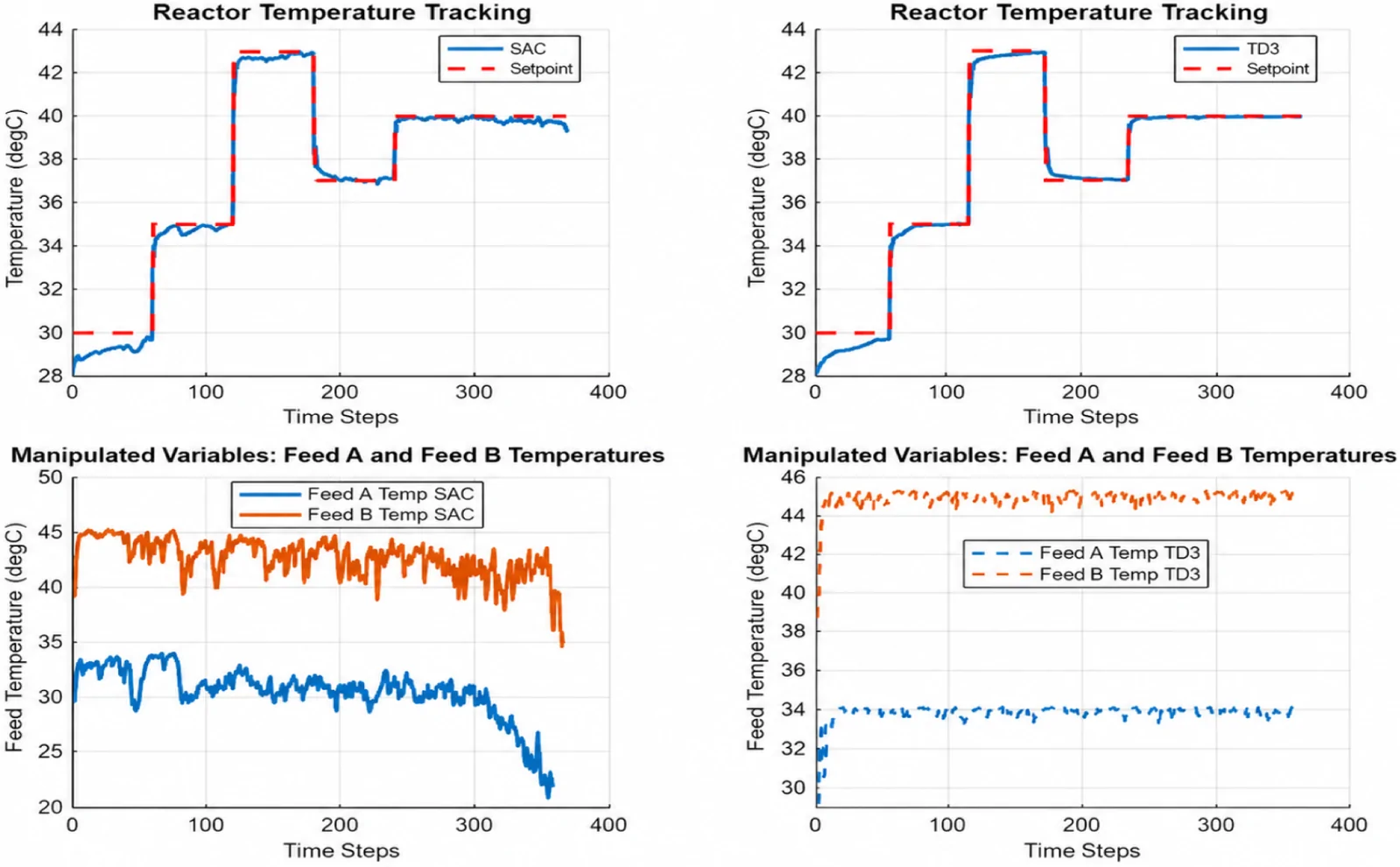
Reactor temperature tracking with manipulated variables as Feed A temperature and Feed B temperature for SAC and TD3 algorithms.

The TD3 controller offers a somewhat smoother and more reliable response when it comes to temperature tracking. Especially throughout the last steady-state area and following the high-temperature transition, the TD3 output closely tracks the set point with less oscillations. Although minor oscillations are seen close to the final operating region, the SAC controller likewise achieves respectable tracking performance. This suggests that whereas TD3 generates a more deterministic closed-loop response, SAC retains a more exploratory control characteristic.

There is a noticeable difference between the two controllers in the manipulated-variable profiles. Both Feed A and Feed B temperatures fluctuate continually over the course of the operational horizon in the SAC scenario. As the simulation comes to a close, Feed A’s temperature progressively drops, and Feed B’s temperature likewise shows discernible swings. This implies that, although at the expense of greater actuator movement, SAC actively employs both modified variables to accomplish set point tracking.

On the other hand, following the initial transient phase, the TD3 manipulated-variable responses are relatively smoother and almost constant. While Feed B’s temperature stays near 45 °C, Feed A’s temperature stays near 34 °C. This demonstrates that TD3 finds a manipulated-variable strategy that is more stable and has less actuator variation.

For the chosen manipulated-variable pair, SAC and TD3 both offer efficient CSTR temperature regulation. While SAC offers adaptive control action with greater oscillatory variation, TD3 exhibits smoother manipulated-variable behavior and somewhat improved tracking stability. Because TD3 accomplishes accurate tracking with less actuator fluctuation, it seems more appropriate for practical application using Feed A temperature and Feed B temperature as controlled variables.

The performance comparison in Table 7 demonstrates that using Feed A and Feed B temperatures as controlled variables, both SAC and TD3 controllers provide efficient reactor temperature tracking. For this manipulated-variable pair, however, TD3 outperforms SAC in terms of total tracking performance. Improved accuracy and decreased cumulative tracking deviation are indicated by the TD3 controller’s lower mean error, MAE, RMSE, ISE, IAE, and best episode error.

**Table 7:** Performance Comparison of SAC and TD3 Controllers for Feed A Temperature and Feed B Temperature

Performance Metric	TD3	SAC
Mean Error (degC)	–0.187	–0.263
Mean Absolute Error, MAE (degC)	0.245	0.314
Root Mean Square Error, RMSE (degC)	0.421	0.454
Integral Square Error, ISE	63.826	74.334
Integral Absolute Error, IAE	88.124	112.952
Maximum Overshoot (degC)	1.549	1.578
Maximum Undershoot (degC)	–2.173	–2.076
Best Episode Error (degC)	0.241	0.282
Training Time (s)	1490.9	1586.9
Actor Feed A Mean Command (%)	99.703	74.617
Actor Feed B Mean Command (%)	99.975	90.215

Both controllers exhibit similar overshoot and undershoot characteristics in terms of transient performance. While SAC provides a somewhat lower maximum undershoot, TD3 offers a slightly lower maximum overshoot than SAC. Both controllers exhibit appropriate transitory behavior during set point changes, as evidenced by the slight difference.

Faster convergence for the chosen CSTR control problem is suggested by the fact that TD3 takes less training time than SAC. While SAC employs comparatively lower average command values, TD3 drives both Feed A and Feed B temperature commands near their upper operational range, according to the mean actor command values. In general, SAC delivers adequate control with relatively lower actuator command levels, while TD3 offers more precise and quicker temperature tracking.

### 6.2 Manipulated Variables—Feed A Temperature, Feed B Flow Rate

Using Feed A temperature and Feed B flow rate as manipulated variables, the comparison demonstrates that both SAC and TD3 properly monitor the stepwise reactor temperature set point. The reactor temperature closely tracks the reference trajectory for the major set point transitions in both scenarios, demonstrating the successful application of the NARX-based environment for closed-loop reinforcement learning control.

The SAC controller tracks temperatures smoothly, with a modest steady-state error following each set point change. Its manipulated-variable response reveals that while Feed B flow rate stays almost constant, Feed A temperature fluctuates moderately over time. This suggests that while Feed B flow rate makes relatively little contribution to the closed-loop adjustment, SAC mostly employs Feed A temperature for corrective control action (Figure 9).

**Figure 9:**
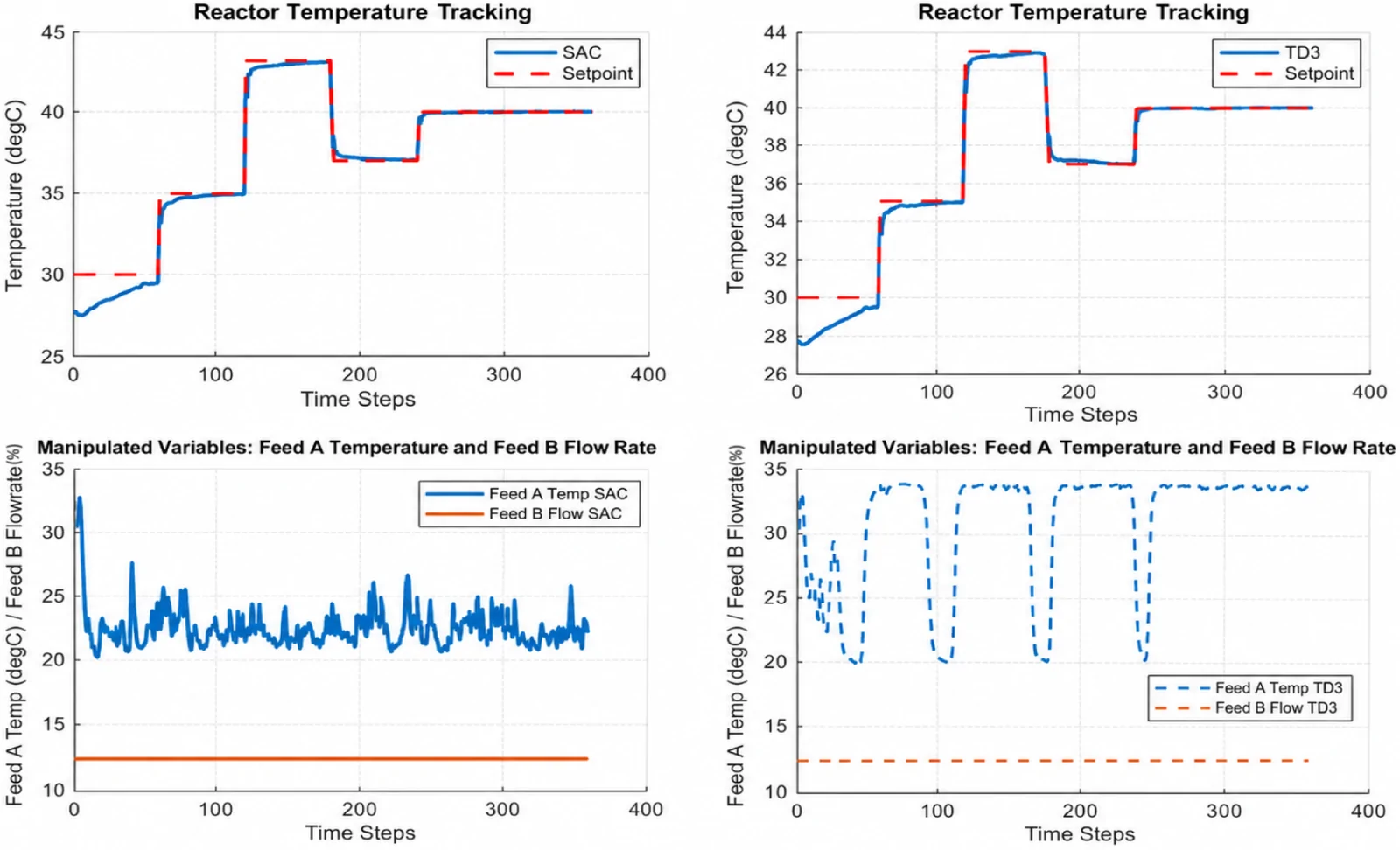
Reactor temperature tracking with manipulated variables as Feed A temperature and Feed B flow rate for SAC and TD3 algorithms.

Additionally, the TD3 controller provides a little more predictable reaction while achieving accurate temperature tracking. While the Feed B flow rate once more stays nearly constant, the Feed A temperature profile in TD3 exhibits frequent fluctuations between higher and lower values. This implies that TD3 has acquired a switching-type or more aggressive Feed A temperature policy.

Both SAC and TD3 offer adequate reactor temperature tracking for the chosen controlled variables, according to the compared results in Table 8. TD3 provides greater tracking accuracy than SAC, as seen by slightly lower MAE, RMSE, ISE, IAE, and best episode error. TD3 also requires less training time, suggesting faster convergence for this case. The mean actor instructions, however, demonstrate that the two controllers employ distinct control strategies: TD3 uses a higher Feed A temperature command with a lower Feed B flow command, while SAC depends more on Feed B flow rate.

**Table 8:** Performance Comparison of SAC and TD3 Controllers for Feed A Temperature and Feed B Flow Rate Control

Performance Metric	SAC	TD3
Mean Error (degC)	–0.317	–0.313
Mean Absolute Error, MAE (degC)	0.373	0.372
Root Mean Square Error, RMSE (degC)	0.677	0.673
Integral Square Error, ISE	164.934	163.071
Integral Absolute Error, IAE	134.172	133.829
Maximum Overshoot (degC)	1.520	1.530
Maximum Undershoot (degC)	–2.482	–2.459
Best Episode Error (degC)	0.369	0.364
Training Time (s)	1728.6	1340.7
Actor Feed A Temp Mean Command (%)	18.640	78.745
Actor Feed B Flow Mean Command (%)	64.202	22.275

### 6.3 Manipulated Variables—Feed B Temperature, Feed A Flow Rate

Considering Feed B temperature and Feed A flow rate as manipulated variables, the comparison demonstrates that both SAC and TD3 achieve efficient reactor temperature tracking for the stepwise set point profile. Both scenarios show that the NARX-based RL environment can allow closed-loop control of the nonlinear CSTR since the reactor temperature closely tracks the set point during the major transitions.

Although the SAC controller’s manipulated-variable response is quite adaptable, it offers smooth temperature tracking. Feed B temperature decreases rapidly from a higher initial value and then remains mostly near the lower operating region, while Feed A flow rate varies significantly over the full time horizon. This suggests that in order to sustain tracking performance, SAC heavily depends on ongoing Feed A flow rate adjustment (Figure 10).

**Figure 10:**
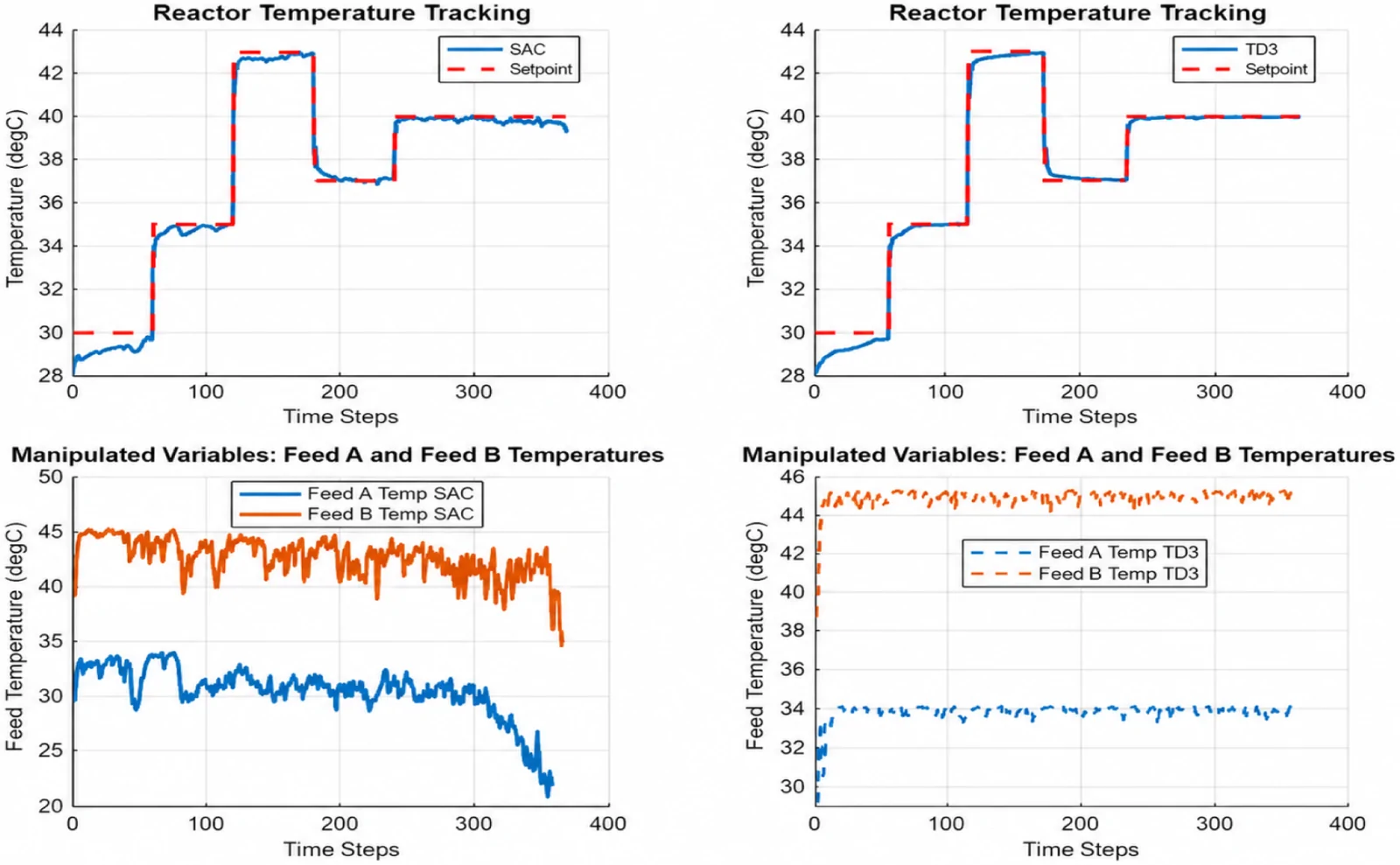
Reactor temperature tracking with manipulated variables as Feed B temperature and Feed A flow rate for SAC and TD3 algorithms.

TD3 controller exhibits a more predictable manipulated-variable pattern and precisely tracks the set point. While Feed A flow rate typically stays at a high level following the initial transient, Feed B temperature varies rapidly at first and then settles near a low value. TD3 employs a more saturated and switching-like control strategy than SAC.

Both controllers offer adequate tracking accuracy overall. But beyond the transitory phase, TD3 achieves similar tracking with a more constant high Feed A flow rate and lower Feed B temperature, while SAC generates a more continuously variable control action. Practically speaking, TD3 provides a more straightforward but saturated control scheme, whereas SAC provides smoother adaptive control.

The performance comparison in Table 9 demonstrates that both SAC and TD3 offer adequate reactor temperature tracking. TD3 provides somewhat greater tracking accuracy than SAC, as seen by slightly lower MAE, RMSE, ISE, IAE, and best episode error. Additionally, TD3 takes less time to train, indicating quicker convergence in this situation. The mean actor commands, however, show distinct control strategies: TD3 heavily depends on a high Feed A flow command with a marginally higher Feed B temperature command, while SAC employs a moderate Feed A flow command with a low Feed B temperature command.

**Table 9:** Performance Comparison of SAC and TD3 Controllers for Feed B Temperature and Feed A Flow Rate Control

Performance Metric	SAC	TD3
Mean Error (degC)	–0.319	–0.322
Mean Absolute Error, MAE (degC)	0.379	0.377
Root Mean Square Error, RMSE (degC)	0.674	0.669
Integral Square Error, ISE	163.651	161.098
Integral Absolute Error, IAE	136.565	135.821
Maximum Overshoot (degC)	1.520	1.529
Maximum Undershoot (degC)	–2.496	–2.503
Best Episode Error (degC)	0.366	0.361
Training Time (s)	1697.5	1476.1
Actor Feed B Temp Mean Command (%)	11.275	16.339
Actor Feed A Flow Mean Command (%)	57.730	95.875

### 6.4 Manipulated Variables—Feed A Flow Rate, Feed B Flow Rate

The comparison demonstrates that both SAC and TD3 can use Feed A flow rate and Feed B flow rate as controlled variables to track the reactor temperature set point. Both controllers follow the stepwise reference trajectory fairly well, according to the upper charts. However, TD3 offers tighter tracking with fewer deviations around the set point, while the SAC response exhibits somewhat larger overshoot and oscillatory behavior throughout the high-temperature area (Figure 11).

**Figure 11:**
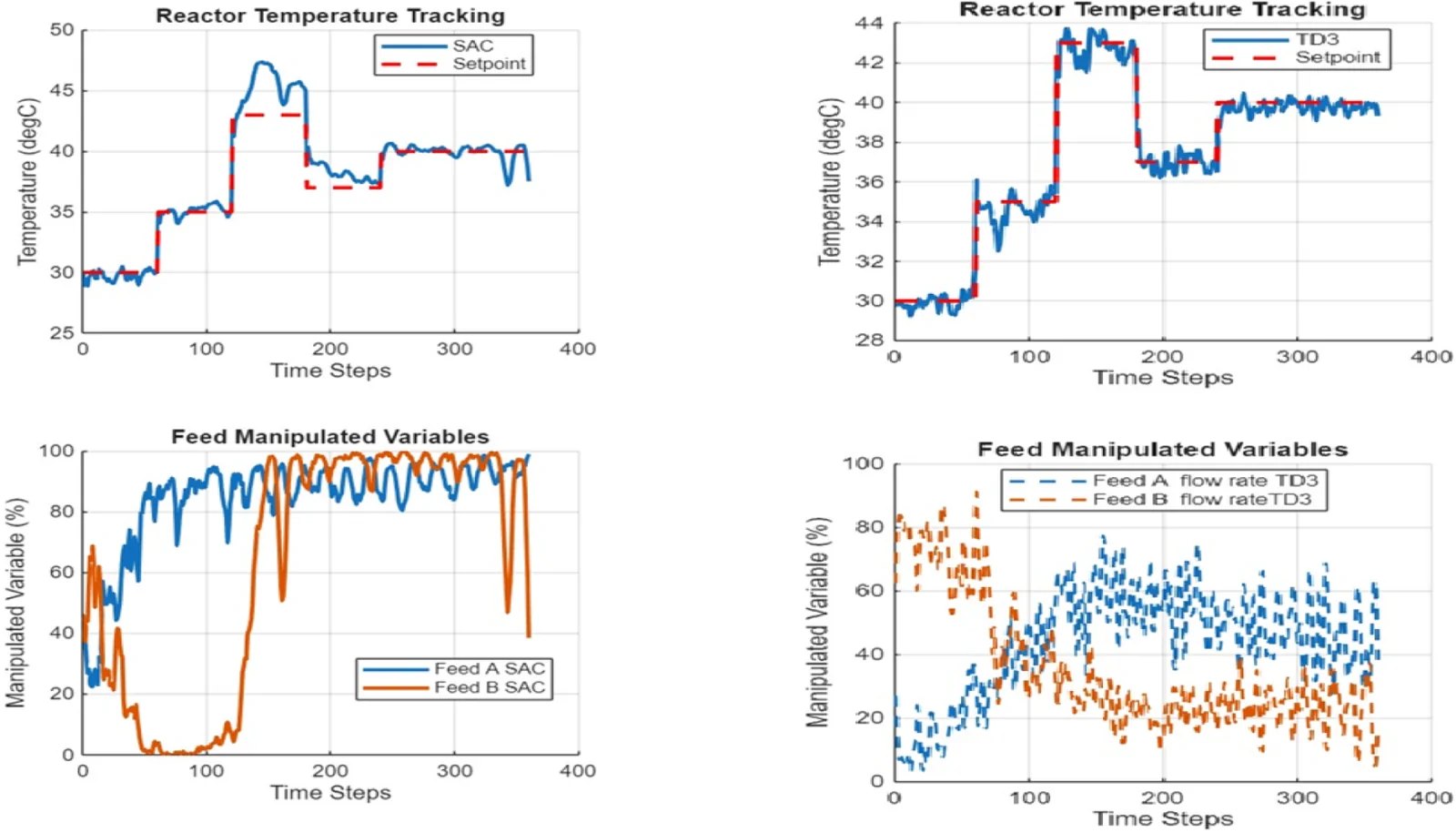
Reactor temperature tracking with manipulated variables as Feed A flow rate and Feed B flow rate for SAC and TD3 algorithms.

Both Feed A and Feed B flow-rate commands often move toward higher command levels after the initial transient in the manipulated-variable plots, where SAC performs more aggressive flow-rate changes. This suggests that SAC achieves temperature tracking with more powerful flow manipulation. TD3, on the other hand, creates a less saturated and more balanced flow-rate profile by distributing the control effort more evenly between Feed A and Feed B flow rates.

Overall, the closed-loop temperature management achieved by both controllers is satisfactory. However, SAC requires more actuator effort to perform tracking, whereas TD3 offers more practical flow-rate fluctuation and relatively smoother temperature tracking. Therefore, from the perspective of tracking accuracy and actuator smoothness, TD3 seems more appropriate for the Feed A flow rate-Feed B flow rate control situation.

The comparison in Table 10 demonstrates that TD3 outperforms SAC in terms of overall tracking performance. Improved tracking accuracy and quicker convergence are indicated by TD3′s lower MAE, RMSE, ISE, IAE, maximum overshoot, and training time. While TD3 performs superior tracking with relatively lower mean flow commands, SAC employs higher average Feed A and Feed B flow-rate commands, indicating greater actuator effort. Therefore, from the perspectives of tracking performance and actuator effort, TD3 is more suited for this manipulated-variable combination.

**Table 10:** Performance Comparison of SAC and TD3 Controllers for Feed A Flow Rate and Feed B Flow Rate Control

Performance Metric	SAC	TD3
Mean Error (degC)	0.554	–0.197
Mean Absolute Error, MAE (degC)	0.868	0.399
Root Mean Square Error, RMSE (degC)	1.355	0.535
Integral Square Error, ISE	660.505	103.053
Integral Absolute Error, IAE	312.531	143.730
Maximum Overshoot (degC)	4.392	1.634
Maximum Undershoot (degC)	–2.778	–2.477
Best Episode Error (degC)	0.277	0.301
Training Time (s)	6844.3	4962.2
Feed A Mean Output (%)	84.526	43.832
Feed B Mean Output (%)	63.083	35.257

### 6.5 Manipulated Variables—Hot Oil Bath, Feed A Temperature

By manipulating the temperature of the Hot Oil Bath and Feed A, both SAC and TD3 are able to track reactor temperature effectively. In all scenarios, the reactor temperature closely follows the stepwise set point, demonstrating that the NARX-based RL control framework can effectively regulate the CSTR temperature (Figure 12).

**Figure 12:**
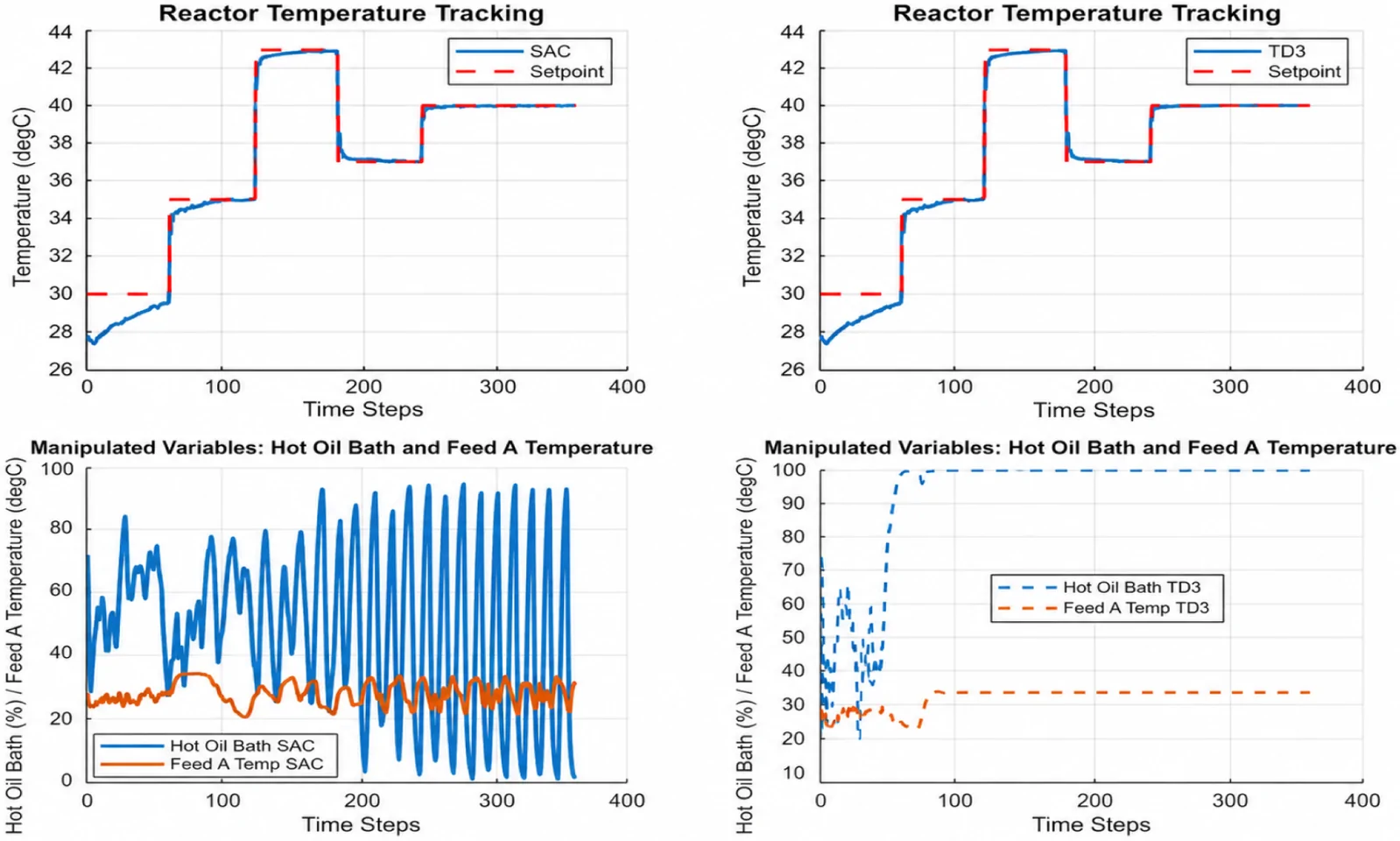
Reactor temperature tracking with manipulated variables as Hot Oil Bath and Feed A temperature for SAC and TD3 algorithms.

In terms of manipulated-variable behavior, Feed A temperature fluctuates within a narrower range, whereas SAC generates a highly oscillatory Hot Oil Bath signal, particularly after the midregion of the response. In contrast, Feed A temperature settles to a virtually constant level whereas TD3 quickly drives the Hot Oil Bath command toward a nearly saturated high value and keeps it there. As a result, TD3 offers a more predictable and saturated control technique, while SAC offers more adaptable yet oscillatory control action.

Both controllers offer satisfactory monitoring overall, while SAC exhibits more actuator variation and TD3 seems to track temperature more smoothly. If reliable tracking is the top priority for practical implementation, TD3 might be better, while SAC might need extra actuator-smoothing or control-rate penalties.

The comparison in Table 11 demonstrates that both SAC and TD3 produce satisfactory reactor temperature monitoring when Hot Oil Bath and Feed A temperature are adjusted variables. TD3 provides somewhat greater tracking accuracy than SAC, as seen by slightly lower MAE, RMSE, ISE, IAE, and best episode error. Additionally, TD3 takes a lot less time to train, indicating quicker convergence. However, SAC accomplishes equivalent tracking with somewhat lower average actuator consumption, while TD3 works with much higher mean Hot Oil Bath and Feed A temperature commands.

**Table 11:** Performance Comparison of SAC and TD3 Controllers for Hot Oil Bath and Feed A Temperature Control

Performance Metric	SAC	TD3
Mean Error (degC)	–0.317	–0.307
Mean Absolute Error, MAE (degC)	0.377	0.363
Root Mean Square Error, RMSE (degC)	0.677	0.668
Integral Square Error, ISE	164.772	160.685
Integral Absolute Error, IAE	135.690	130.802
Maximum Overshoot (degC)	1.510	1.540
Maximum Undershoot (degC)	–2.585	–2.554
Best Episode Error (degC)	0.364	0.362
Training Time (s)	2977.5	1353.3
Actor Hot Oil Bath Mean Command (%)	49.518	93.244
Actor Feed A Temperature Mean Command (%)	54.108	89.223

### 6.6 Manipulated Variables—Hot Oil Bath, Feed B Temperature

By manipulating the temperature of the Hot Oil Bath and Feed B, both SAC and TD3 are able to provide adequate reactor temperature tracking. In all scenarios, the reactor temperature closely tracks the stepwise set point, suggesting that the NARX-based RL environment is appropriate for closed-loop CSTR temperature control (Figure 13).

**Figure 13:**
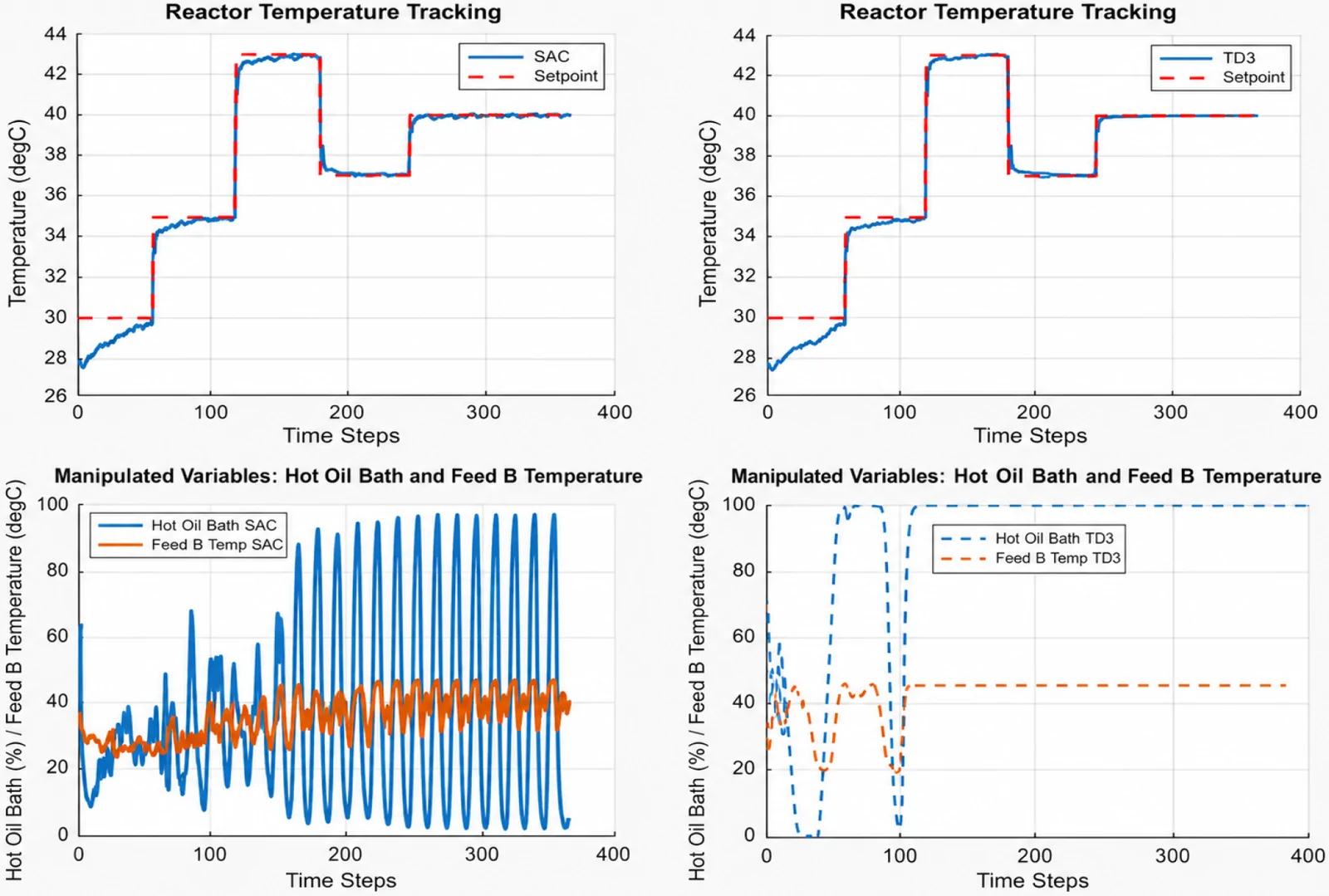
Reactor temperature tracking with manipulated variables as Hot Oil Bath and Feed B temperature for SAC and TD3 algorithms.

Feed B temperature fluctuates moderately around a reduced temperature range, while SAC generates a highly oscillatory Hot Oil Bath signal for the controlled variables, particularly after the midpoint of the operational horizon. On the other hand, Feed B temperature settles to a virtually constant value following the initial transient, while TD3 quickly drives the Hot Oil Bath command toward a nearly saturated high value and maintains it.

Overall, SAC offers more adaptive yet oscillatory control action, while TD3 offers a more predictable and stable manipulated-variable profile. Practically, TD3 is better because of its lower fluctuation, but SAC might need more smoothing or control-rate penalties to lessen actuator oscillations. The comparison in Table 12 demonstrates that using the temperature of the Hot Oil Bath and Feed B as controlled variables, both SAC and TD3 offer adequate reactor temperature tracking. TD3 provides somewhat greater tracking accuracy than SAC, as seen by slightly lower MAE, RMSE, ISE, IAE, and best episode error. Additionally, TD3 requires less training time, which implies quicker convergence. However, SAC accomplishes similar tracking with lower mean actuator effort, while TD3 requires much greater average Hot Oil Bath and Feed B temperature commands.

**Table 12:** Performance Comparison of SAC and TD3 Controllers for Hot Oil Bath and Feed B Temperature Control

Performance Metric	SAC	TD3
Mean Error (degC)	–0.317	–0.307
Mean Absolute Error, MAE (degC)	0.389	0.372
Root Mean Square Error, RMSE (degC)	0.684	0.675
Integral Square Error, ISE	168.345	164.257
Integral Absolute Error, IAE	140.161	133.757
Maximum Overshoot (degC)	1.464	1.559
Maximum Undershoot (degC)	–2.661	–2.565
Best Episode Error (degC)	0.365	0.362
Training Time (s)	1549.4	1173.6
Actor Hot Oil Bath Mean Command (%)	34.673	86.407
Actor Feed B Temperature Mean Command (%)	53.345	88.421

### 6.7 Manipulated Variables—Hot Oil Bath, Feed A Flow Rate

By manipulating the Hot Oil Bath and Feed A flow rate, both SAC and TD3 are able to provide satisfactory reactor temperature tracking. In all scenarios, the reactor temperature closely tracks the stepwise set point, demonstrating efficient closed-loop control of the CSTR temperature (Figure 14).

**Figure 14:**
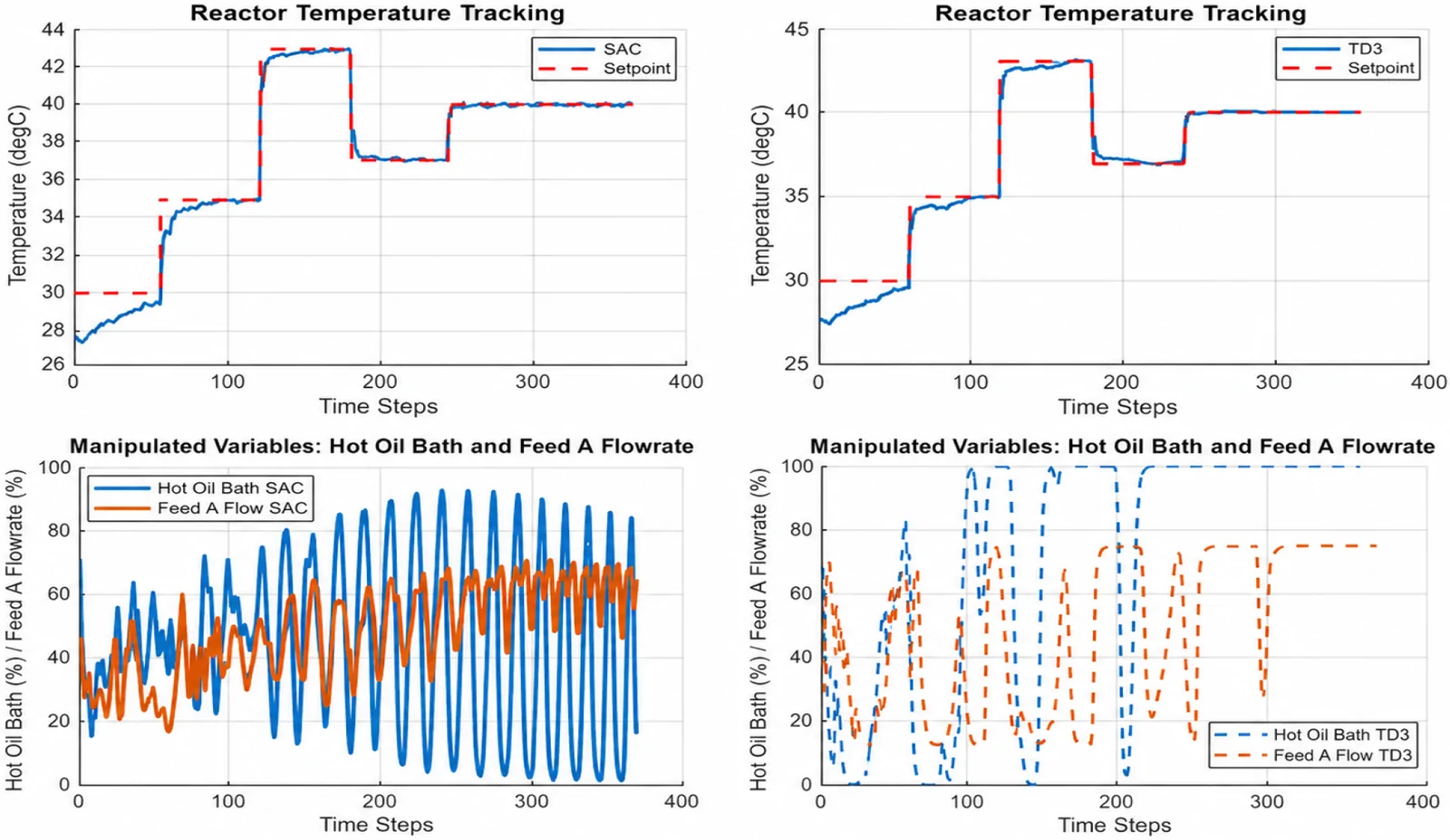
Reactor temperature tracking with manipulated variables as Hot Oil Bath and Feed A flow rate for SAC and TD3 algorithms.

While Feed A flow rate grows smoothly with mild changes, SAC exhibits highly oscillatory Hot Oil Bath action for the controlled variables, particularly after the middle of the reaction. TD3, on the other hand, exhibits more switching-type manipulated-variable behavior, with both Feed A flow rate and Hot Oil Bath moving abruptly between low and high command levels. While SAC offers a smoother temperature response but more intense actuator oscillations, TD3 generally provides slightly more predictable tracking. Practically speaking, both controllers work well; however, the manipulated-variable profiles would benefit from additional actuator-smoothing or control-move penalties.

The comparison in Table 13 shows that using the Hot Oil Bath and Feed A flow rate as controlled variables, both SAC and TD3 produce satisfactory reactor temperature tracking. SAC provides somewhat greater tracking accuracy than TD3, as evidenced by slightly lower MAE, RMSE, ISE, IAE, and maximum overshoot. On the other hand, TD3 offers a slightly lower best episode error and requires significantly less training time, indicating faster convergence. While both controllers employ virtually identical Feed A flow rate commands, the mean command values indicate that TD3 utilizes a greater Hot Oil Bath command.

**Table 13:** Performance Comparison of SAC and TD3 Controllers for Hot Oil Bath and Feed A Flow Rate Control

Performance Metric	SAC	TD3
Mean Error (degC)	–0.316	–0.307
Mean Absolute Error, MAE (degC)	0.378	0.400
Root Mean Square Error, RMSE (degC)	0.679	0.693
Integral Square Error, ISE	165.918	173.136
Integral Absolute Error, IAE	136.223	143.931
Maximum Overshoot (degC)	1.579	1.595
Maximum Undershoot (degC)	–2.571	–2.563
Best Episode Error (degC)	0.362	0.356
Training Time (s)	3064.4	1187.5
Actor Hot Oil Bath Mean Command (%)	43.754	70.644
Actor Feed A Flow rate Mean Command (%)	58.730	58.529

### 6.8 Manipulated Variables—Hot Oil Bath, Feed B Flow Rate

Using the Hot Oil Bath and Feed B flow rate as controlled variables, both SAC and TD3 offer efficient reactor temperature tracking. With minimal steady-state error following each transition, the reactor temperature in both scenarios closely tracks the stepwise set point, demonstrating adequate closed-loop control capability (Figure 15).

**Figure 15:**
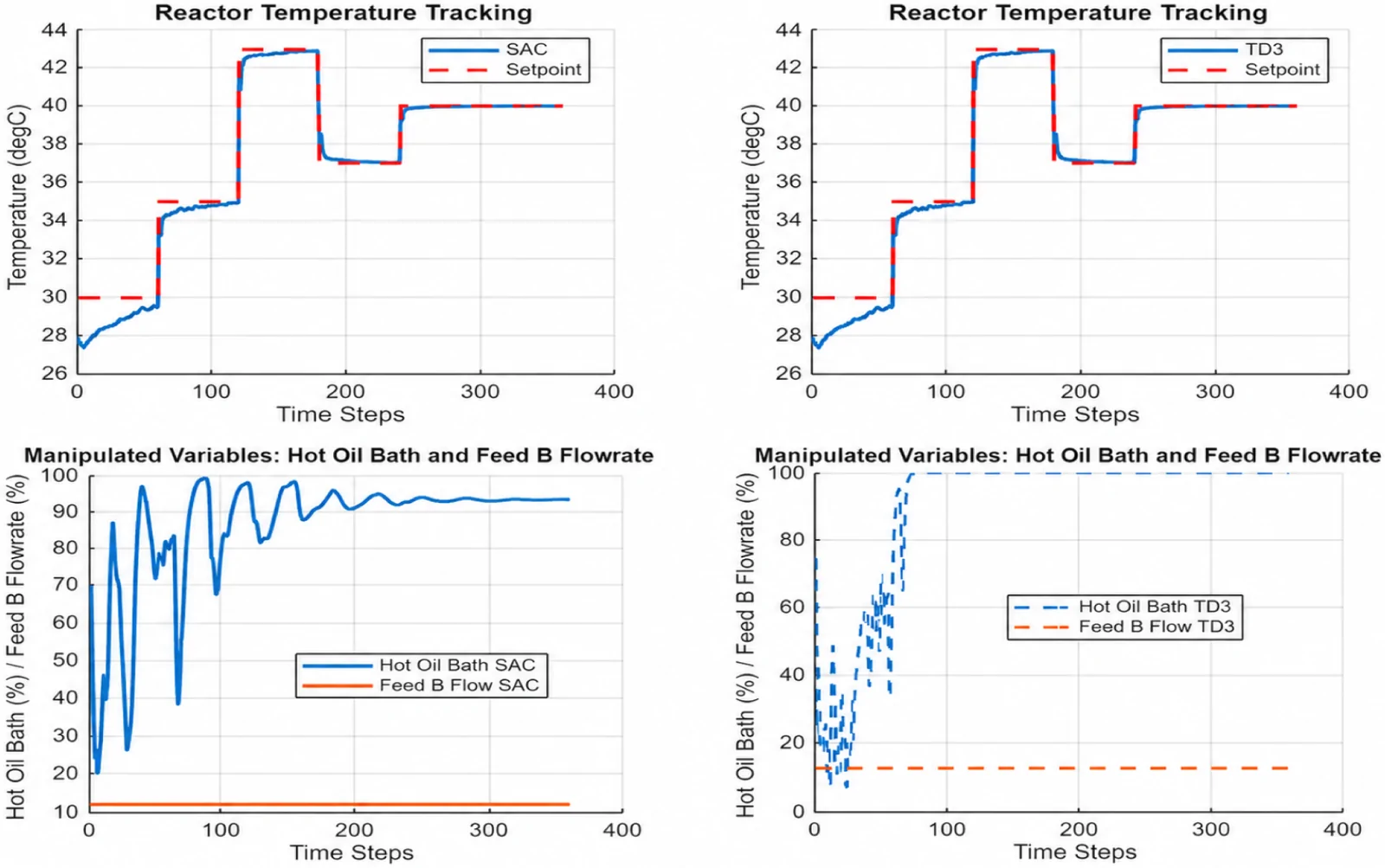
Reactor temperature tracking with manipulated variables as Hot Oil Bath and Feed B flow rate for SAC and TD3 algorithms.

While the Feed B flow rate stays nearly constant at a low level, SAC displays an initially oscillating Hot Oil Bath command for the manipulated-variable response that eventually settles near a high value. The Hot Oil Bath command is likewise driven toward saturation by TD3, but after the initial transient, it rises more quickly and exhibits more deterministic behavior. For TD3, the Feed B flow rate is essentially unchanged.

Overall, the primary manipulated variable used by both controllers is Hot Oil Bath, with Feed B flow rate having a negligible impact on control action. After early oscillations, SAC offers a smoother settling pattern, while TD3 offers a more direct and saturated control response. Practically speaking, SAC exhibits a little more gradual control adjustment, whereas TD3 provides faster actuator convergence. The comparison in Table 14 highlights that utilizing the Hot Oil Bath and Feed B flow rate as controlled variables, both SAC and TD3 obtain nearly identical reactor temperature tracking performance. TD3 shows somewhat superior tracking accuracy with slightly lower RMSE, ISE, IAE, and best episode error. Additionally, TD3 requires less training time than SAC, indicating faster convergence. However, SAC achieves similar tracking with comparatively lower mean actuator effort, whereas TD3 requires higher average Hot Oil Bath and Feed B flow rate commands.

**Table 14:** Performance Comparison of SAC and TD3 Controllers for Hot Oil Bath and Feed B Flow Rate Control

Performance Metric	SAC	TD3
Mean Error (degC)	–0.318	–0.318
Mean Absolute Error, MAE (degC)	0.372	0.372
Root Mean Square Error, RMSE (degC)	0.671	0.668
Integral Square Error, ISE	162.243	160.480
Integral Absolute Error, IAE	133.941	133.865
Maximum Overshoot (degC)	1.510	1.523
Maximum Undershoot (degC)	–2.597	–2.661
Best Episode Error (degC)	0.371	0.365
Training Time (s)	1986.9	1421.9
Actor Hot Oil Bath Mean Command (%)	87.401	90.218
Actor Feed B Flow Rate Mean Command (%)	59.143	93.289

## 7 Conclusion and Future Scope

This study addressed a significant gap in the Continuous Stirred Tank Reactor (CSTR) control literature by focusing on intelligent temperature regulation in a highly nonlinear and exothermic reactor environment. Although earlier studies have independently demonstrated the effectiveness of model-based control, data-driven modeling, and reinforcement learning, their combined application to a practical lab-scale CSTR remains comparatively limited. Many existing approaches depend heavily on accurate first-principles models, simulation-only validation, or restricted operating conditions, which limits their adaptability and real-time applicability. This motivated the development of a more flexible, data-supported, and control-oriented reinforcement learning framework for CSTR temperature regulation.

To bridge this gap, a progressive modeling and control strategy was adopted. First-principles modeling was initially used to describe the fundamental mass and energy balance dynamics of the reactor and to establish the thermal behavior of the process. This was followed by gray-box and nonlinear data-driven modeling approaches, culminating in a high-fidelity NARX-based model capable of predicting the reactor temperature response under different manipulated-variable conditions. The NARX model served as a practical environment for reinforcement learning controller training, thereby reducing the direct dependence on a purely mechanistic model while retaining the nonlinear input–output behavior of the experimental CSTR system.

Twin Delayed Deep Deterministic Policy Gradient (TD3) and Soft Actor-Critic (SAC), two sophisticated deep reinforcement learning algorithms, were studied for continuous manipulated-variable control. To assess the closed-loop temperature tracking capacity, several combinations of manipulated factors, such as feed flow rates, feed temperatures, and hot oil bath input, were taken into consideration. The findings shown that SAC and TD3 could both effectively control the reactor temperature for the stepwise set point trajectory. While TD3 offered more deterministic control behavior with consistent convergence features, SAC showed considerable exploratory activity and adaptive control action.

From the comparative analysis, it was observed that the TD3-based controller produced slightly superior tracking indices for most of the manipulated-variable combinations considered. In several cases, TD3 achieved lower values of MAE, RMSE, ISE, IAE, and training time, indicating improved tracking accuracy and faster convergence. SAC also provided acceptable closed-loop performance, but its manipulated-variable profiles were generally more oscillatory because of the stochastic nature of the policy and entropy-driven exploration. Therefore, while both reinforcement learning controllers were effective, the overall trend suggests that TD3 offered a more balanced compromise between tracking accuracy, convergence speed, and control stability for the present CSTR temperature regulation problem.

Overall, the findings show that combining control-oriented nonlinear modeling with reinforcement learning is a potential approach to practical CSTR temperature management. The suggested methodology lowers reliance on solely mechanistic models, increases adaptability to disturbances and uncertainty, and facilitates the move from simulation-oriented studies to real-time deployable control structures. As a result, this study reinforces the argument for reinforcement learning-based temperature management of lab-scale Continuous Stirred Tank Reactors as a timely and relevant direction in advanced process control research.

Building on the current work, future studies can concentrate on expanding the suggested reinforcement learning framework to more comprehensive, adaptable, and practically applicable reactor control scenarios. With a focus on sample efficiency, convergence speed, robustness under severe disturbances, and final control performance, additional comparisons between TD3 and SAC and other sophisticated deep reinforcement learning algorithms, such as Proximal Policy Optimization (PPO), Deep Deterministic Policy Gradient (DDPG), and constrained reinforcement learning techniques, may be conducted. A multivariable control framework that simultaneously regulates reactor temperature and reactant concentration using a variety of manipulated inputs, including feed flow rates, feed temperatures, coolant flow rates, and heating/cooling utility signals, can be developed from the current single-variable temperature regulation strategy.

Moving from simulation to real and long-term implementation opens up further possibilities. Hardware-in-the-loop implementation on industrial platforms such as PLCs or edge devices can be used to assess real-time inference, latency, and compatibility with current control infrastructure. The framework can also be extended to include batch and semibatch reactors, online transfer learning for adjusting to catalyst deactivation, fouling, and sensor drift, decentralized control of interconnected reactor networks, and the incorporation of physics-informed neural networks to improve interpretability and reduce data requirements. Future research should focus on safety-critical RL with formal constraint handling and fail-safe mechanisms, as well as promoting long-term industrial viability through energy-aware rewards, model compression, transfer learning, predictive maintenance, and low-power edge deployment Table 15 .

**Table 15:** Input-to-Hidden Weight Matrix *W*_1*D*_ (20 × 90)

*W*_1*D*_: Columns 1–10										
Row	1	2	3	4	5	6	7	8	9	10
1	0.0597	–0.0789	–0.0587	0.0473	0.0648	0.1188	0.0632	–0.0681	–0.0578	–0.0103
2	–0.0891	–0.0245	–0.0064	–0.0265	0.0040	0.0937	–0.0854	–0.0190	–0.0035	0.0199
3	0.0113	0.0638	0.0035	–0.0354	–0.0547	0.0101	0.0067	0.0426	–0.0004	–0.0233
4	0.0024	–0.0026	0.0374	–0.0114	–0.0216	–0.0566	0.0045	0.0139	0.0397	0.0055
5	–0.0500	–0.0279	–0.0178	–0.0676	–0.0028	0.0420	–0.0485	0.0007	–0.0219	–0.0396
6	–0.0355	–0.0560	–0.0128	0.0012	–0.0890	0.0819	–0.0364	–0.0214	–0.0089	–0.0236
7	–0.0899	–0.0672	0.0966	0.0295	0.0272	–0.0122	–0.0831	–0.0482	0.0964	0.0128
8	0.0607	–0.0199	–0.0199	0.1024	–0.0803	0.0805	0.0573	–0.0259	–0.0110	0.0647
9	0.0448	0.0598	–0.0581	–0.1124	0.0820	–0.0678	0.0422	0.0375	–0.0612	–0.1054
10	–0.0215	0.1059	0.0222	0.0285	0.0350	0.0470	–0.0244	0.0812	0.0240	–0.0243
11	–0.0226	0.0855	0.0045	0.0408	0.0621	–0.0060	–0.0263	0.0565	0.0020	0.0497
12	0.0287	–0.0000	–0.0237	0.0348	0.0204	–0.1139	0.0271	–0.0200	–0.0215	0.0014
13	–0.0467	0.0543	0.0257	–0.0123	–0.0030	–0.0092	–0.0474	0.0294	0.0282	–0.0923
14	–0.0609	–0.0339	–0.0390	0.0468	0.0523	–0.0127	–0.0599	–0.0269	–0.0458	0.0216
15	–0.0198	–0.0098	0.0725	–0.0575	0.0411	0.0158	–0.0149	–0.0048	0.0830	–0.0660
16	0.0568	–0.0480	0.1000	0.0157	0.0322	–0.0950	0.0542	–0.0316	0.0891	–0.0263
17	–0.0066	–0.0482	0.0337	0.0673	–0.0655	0.0209	–0.0103	–0.0643	0.0383	0.0472
18	–0.0728	0.0626	0.0172	–0.0957	–0.0022	0.0056	–0.0737	0.0819	0.0206	0.0037
19	0.0904	–0.0521	–0.1353	0.0603	0.0633	–0.1448	0.0937	–0.0463	–0.1250	0.0684
20	0.0119	0.0348	0.0579	–0.1291	–0.1215	0.0946	0.0101	0.0089	0.0608	–0.1770

*W*_1*D*_: Columns 11–20										
Row	11	12	13	14	15	16	17	18	19	20
1	0.0347	0.1050	0.0642	–0.0613	–0.0588	–0.0194	0.0307	0.0670	0.0660	–0.0440
2	–0.0418	0.0839	–0.0788	–0.0133	0.0094	0.0286	–0.0548	0.0745	–0.0743	–0.0078
3	–0.0266	0.0056	0.0016	0.0304	–0.0097	0.0081	0.0007	0.0081	–0.0027	0.0086
4	–0.0007	–0.0752	0.0112	0.0278	0.0601	–0.0830	–0.0303	–0.0646	0.0159	0.0425
5	–0.0240	0.0781	–0.0485	0.0082	–0.0139	–0.0604	–0.0665	0.1016	–0.0462	0.0383
6	0.0087	0.0673	–0.0342	–0.0012	0.0018	–0.0235	0.0083	0.0606	–0.0324	0.0323
7	–0.0549	–0.0011	–0.0747	–0.0390	0.1129	–0.0169	–0.0889	–0.0377	–0.0674	–0.0140
8	–0.0331	0.0695	0.0576	–0.0231	–0.0167	0.1139	–0.0093	0.0600	0.0570	–0.0295
9	0.0755	–0.0714	0.0387	0.0323	–0.0784	–0.0410	0.1091	–0.0492	0.0342	0.0143
10	0.0515	0.0367	–0.0290	0.0643	0.0136	0.0019	0.0778	0.0508	–0.0330	0.0371
11	0.0250	–0.0073	–0.0323	0.0415	–0.0090	0.0417	0.0376	0.0048	–0.0368	0.0161
12	–0.0071	–0.0814	0.0218	–0.0296	–0.0341	0.0109	–0.0116	–0.0557	0.0162	–0.0500
13	–0.0355	–0.0407	–0.0517	0.0143	0.0250	–0.0924	–0.0262	–0.0129	–0.0564	–0.0032
14	–0.0525	0.0130	–0.0583	–0.0250	–0.0370	0.0044	–0.0680	0.0072	–0.0564	–0.0127
15	0.0008	0.0350	–0.0085	0.0014	0.0978	–0.0134	–0.0209	0.0005	–0.0052	0.0093
16	0.0572	–0.0788	0.0522	–0.0254	0.0820	–0.0381	0.0558	–0.0788	0.0530	–0.0082
17	0.0047	–0.0096	–0.0108	–0.0701	0.0361	0.0905	0.0105	0.0160	–0.0141	–0.0840
18	0.0586	–0.0238	–0.0694	0.0872	0.0349	–0.0012	0.0418	–0.0051	–0.0671	0.1010
19	0.0087	–0.1075	0.0996	–0.0436	–0.1150	0.0734	–0.0220	–0.1207	0.1044	–0.0359
20	–0.1070	0.0973	0.0055	–0.0008	0.0410	–0.0703	–0.0715	0.0753	0.0015	–0.0181

*W*_1*D*_: Columns 21–30										
Row	21	22	23	24	25	26	27	28	29	30
1	–0.0677	–0.0399	0.0676	0.0513	0.0720	–0.0360	–0.0810	–0.0283	0.1211	0.0923
2	0.0015	0.0425	–0.0162	0.0608	–0.0683	–0.0039	–0.0071	0.0240	0.0316	0.0760
3	–0.0009	–0.0254	–0.0152	0.0303	–0.0118	–0.0050	0.0112	–0.0148	–0.0501	–0.0080
4	0.0573	–0.1380	–0.0261	–0.0443	0.0239	0.0504	0.0546	–0.1520	0.0077	0.0186
5	–0.0187	0.0215	–0.0751	0.0747	–0.0387	0.0576	–0.0416	0.0511	–0.0437	0.0623
6	–0.0042	0.0766	–0.0035	0.0221	–0.0261	0.0540	–0.0190	0.0715	0.0399	0.0032
7	0.1003	–0.0102	–0.0411	–0.0816	–0.0536	–0.0004	0.0780	–0.0238	0.0285	0.0118
8	–0.0101	0.1087	–0.0177	0.0673	0.0552	–0.0306	0.0013	0.1364	–0.0408	0.0708
9	–0.0760	–0.0425	0.0930	–0.0001	0.0254	0.0015	–0.0661	0.0229	0.0556	–0.0566
10	0.0225	–0.0422	0.0351	0.0944	–0.0417	0.0204	0.0451	–0.0428	–0.0289	0.0291
11	0.0056	–0.0155	0.0141	0.0187	–0.0478	0.0018	0.0283	–0.0412	–0.0441	–0.0273
12	–0.0228	–0.0328	–0.0543	–0.0087	0.0070	–0.0594	0.0023	–0.0243	–0.1413	0.0056
13	0.0288	–0.1119	–0.0196	0.0280	–0.0645	–0.0115	0.0529	–0.0461	–0.0226	–0.0407
14	–0.0416	0.0453	–0.0232	–0.0146	–0.0527	–0.0024	–0.0495	0.0584	–0.0034	0.0088
15	0.0832	0.0135	0.0191	–0.0587	0.0023	0.0136	0.0672	–0.0044	0.0900	–0.0140
16	0.0751	–0.0309	0.0452	–0.0880	0.0549	0.0064	0.0611	–0.0358	0.0450	–0.0946
17	0.0380	0.0628	–0.0364	0.0542	–0.0200	–0.0877	0.0430	0.0378	–0.0652	–0.0102
18	0.0232	0.0558	0.0354	0.0035	–0.0604	0.1079	0.0082	0.0187	0.0713	0.0021
19	–0.1180	0.0888	0.0010	–0.1619	0.1121	–0.0292	–0.1252	0.0769	0.0209	–0.0827
20	0.0482	–0.0285	–0.0817	0.0776	–0.0071	–0.0274	0.0599	0.0521	–0.1034	0.0280

*W*_1*D*_: Columns 31–40										
Row	31	32	33	34	35	36	37	38	39	40
1	0.0744	–0.0260	–0.0879	–0.0693	0.1277	0.0718	0.0762	–0.0163	–0.0863	–0.0401
2	–0.0673	–0.0064	–0.0155	0.0162	0.0042	0.0678	–0.0658	–0.0037	–0.0132	0.0036
3	–0.0143	–0.0088	0.0165	0.0241	0.0124	–0.0461	–0.0172	–0.0126	0.0076	0.0057
4	0.0249	0.0447	0.0504	–0.1052	–0.0239	0.0518	0.0300	0.0439	0.0634	–0.0699
5	–0.0365	0.0593	–0.0504	0.0405	–0.0791	0.1125	–0.0318	0.0590	–0.0387	0.0585
6	–0.0269	0.0578	–0.0219	0.0240	0.0246	0.0709	–0.0217	0.0540	–0.0102	0.0061
7	–0.0496	0.0033	0.0666	–0.0837	–0.0414	0.0617	–0.0444	0.0151	0.0722	–0.0785
8	0.0525	–0.0280	0.0123	0.1323	–0.0052	0.0408	0.0516	–0.0276	0.0067	0.1015
9	0.0225	0.0068	–0.0582	0.0989	0.1263	–0.1018	0.0172	0.0099	–0.0698	0.1088
10	–0.0419	0.0169	0.0497	–0.0328	0.0439	–0.0365	–0.0469	0.0130	0.0406	–0.0219
11	–0.0504	0.0035	0.0353	–0.0941	0.0174	–0.0805	–0.0554	0.0050	0.0377	–0.1382
12	0.0040	–0.0584	0.0137	–0.0139	–0.1089	–0.0180	–0.0006	–0.0576	0.0113	–0.0061
13	–0.0643	–0.0070	0.0595	–0.0132	0.0284	–0.0734	–0.0709	0.0016	0.0572	0.0158
14	–0.0522	–0.0038	–0.0610	0.0454	–0.0291	0.0294	–0.0511	0.0015	–0.0550	0.0583
15	0.0038	0.0189	0.0636	–0.0466	0.0567	–0.0080	0.0053	0.0271	0.0586	–0.0638
16	0.0523	0.0038	0.0539	–0.0699	0.0751	–0.0811	0.0563	0.0009	0.0539	–0.0773
17	–0.0218	–0.0891	0.0483	0.0227	–0.0166	–0.0276	–0.0251	–0.0871	0.0400	0.0078
18	–0.0598	0.1034	0.0093	–0.0177	–0.0039	0.0724	–0.0554	0.0951	0.0159	–0.0244
19	0.1117	–0.0269	–0.1258	0.0494	–0.0608	–0.0538	0.1148	–0.0196	–0.1209	0.0443
20	–0.0099	–0.0218	0.0726	0.1089	–0.0174	–0.0464	–0.0165	–0.0214	0.0588	0.1366

*W*_1*D*_: Columns 41–50										
Row	41	42	43	44	45	46	47	48	49	50
1	0.0684	0.0131	0.0772	–0.0098	–0.0801	0.0155	0.0591	0.0175	0.0772	–0.0076
2	–0.0063	–0.0042	–0.0660	–0.0117	–0.0136	0.0115	0.0268	–0.0484	–0.0680	–0.0125
3	0.0426	0.0321	–0.0179	–0.0082	–0.0047	–0.0437	0.0065	0.0631	–0.0144	0.0024
4	–0.1034	0.0364	0.0312	0.0277	0.0731	–0.0338	–0.0908	0.0207	0.0308	0.0138
5	–0.0637	0.0260	–0.0277	0.0566	–0.0268	0.1035	–0.0132	–0.0042	–0.0334	0.0467
6	–0.0054	0.0318	–0.0196	0.0410	0.0075	0.0870	0.0256	0.0186	–0.0277	0.0252
7	–0.0801	–0.0476	–0.0418	0.0118	0.0769	–0.0349	–0.0632	–0.0268	–0.0432	0.0100
8	–0.0140	0.0646	0.0509	–0.0247	0.0122	0.0600	–0.0229	0.0655	0.0521	–0.0177
9	0.1070	0.0007	0.0145	0.0218	–0.0812	0.0622	0.0263	0.0188	0.0179	0.0390
10	0.0563	0.0249	–0.0505	0.0240	0.0244	–0.0220	0.0084	0.0211	–0.0466	0.0351
11	0.0295	0.0104	–0.0581	0.0176	0.0279	–0.1246	–0.0160	0.0301	–0.0551	0.0287
12	–0.0565	0.0608	–0.0021	–0.0429	0.0085	–0.0170	–0.0468	0.0763	0.0030	–0.0306
13	0.0533	0.0420	–0.0740	0.0231	0.0455	–0.0064	0.0553	0.0332	–0.0677	0.0370
14	–0.0214	–0.0289	–0.0522	–0.0003	–0.0652	0.0522	–0.0356	–0.0162	–0.0552	–0.0016
15	0.0429	–0.0986	0.0069	0.0236	0.0591	–0.0197	0.0559	–0.0999	0.0052	0.0295
16	0.0651	–0.1139	0.0578	–0.0028	0.0561	–0.0730	0.0482	–0.0988	0.0510	–0.0097
17	–0.0098	0.0477	–0.0274	–0.0814	0.0320	0.0227	–0.0004	0.0194	–0.0273	–0.0699
18	–0.0240	0.0604	–0.0531	0.0804	0.0252	0.0684	0.0105	0.0248	–0.0568	0.0648
19	–0.0593	–0.1484	0.1162	–0.0184	–0.1066	0.0944	–0.0304	–0.1532	0.1155	–0.0157
20	0.0150	–0.0034	–0.0183	–0.0076	0.0559	0.0768	–0.0269	0.0226	–0.0152	0.0096

*W*_1*D*_: Columns 51–60										
Row	51	52	53	54	55	56	57	58	59	60
1	–0.0759	0.1098	0.0588	0.0554	0.0759	–0.0059	–0.0671	0.0911	0.0725	0.0657
2	–0.0168	–0.0085	0.0630	–0.0489	–0.0677	–0.0185	–0.0136	–0.0504	0.0474	–0.0382
3	–0.0108	–0.0588	–0.0130	0.0294	–0.0150	0.0125	–0.0255	–0.0152	0.0090	0.0016
4	0.0816	0.0248	–0.0513	0.0452	0.0325	–0.0043	0.0894	–0.0121	–0.0755	0.0647
5	–0.0228	0.1051	0.0007	0.0114	–0.0319	0.0354	–0.0013	0.0214	0.0017	0.0485
6	0.0225	0.1299	0.0159	0.0486	–0.0272	0.0066	0.0399	0.0568	0.0006	0.0954
7	0.0844	–0.0300	–0.0594	0.0765	–0.0416	0.0041	0.1005	–0.0588	–0.0962	0.1321
8	0.0185	0.0431	–0.0299	0.0555	0.0506	–0.0139	0.0059	0.0621	–0.0065	0.0312
9	–0.0873	0.0820	–0.0364	–0.0403	0.0151	0.0539	–0.1072	0.0843	–0.0266	–0.0515
10	0.0117	–0.0283	–0.0382	–0.0628	–0.0477	0.0504	–0.0083	0.0410	–0.0239	–0.1003
11	0.0221	–0.0933	–0.0322	–0.0076	–0.0564	0.0411	0.0125	0.0297	–0.0047	–0.0431
12	0.0130	0.0313	–0.0607	0.0456	0.0025	–0.0163	0.0027	0.0822	–0.0221	–0.0126
13	0.0468	0.0198	0.0375	–0.0253	–0.0699	0.0530	0.0310	0.0835	0.0552	–0.0545
14	–0.0750	0.0302	–0.0485	0.0073	–0.0567	–0.0066	–0.0675	–0.0186	–0.0606	0.0206
15	0.0612	0.0354	0.0738	–0.0312	0.0040	0.0291	0.0656	0.0129	0.0515	–0.0067
16	0.0556	–0.0607	0.0183	–0.0865	0.0503	–0.0195	0.0616	–0.1366	0.0323	–0.0711
17	0.0317	0.0880	–0.0075	–0.0487	–0.0286	–0.0651	0.0176	0.1243	0.0137	–0.0684
18	0.0367	0.0409	0.0196	0.0264	–0.0556	0.0487	0.0467	0.0295	–0.0242	0.0689
19	–0.0881	0.0798	0.0122	–0.0808	0.1178	–0.0172	–0.0826	0.0849	–0.0097	–0.0698
20	0.0457	0.0341	–0.0582	–0.0090	–0.0170	0.0256	0.0273	0.0426	–0.0281	–0.0227

*W*_1*D*_: Columns 61–70										
Row	61	62	63	64	65	66	67	68	69	70
1	0.0724	–0.0032	–0.0664	0.1092	0.0809	0.0604	0.0758	–0.0086	–0.0712	0.0635
2	–0.0683	–0.0202	–0.0139	–0.0503	0.0544	–0.0678	–0.0685	–0.0217	–0.0336	–0.0677
3	–0.0124	0.0135	–0.0317	–0.0437	–0.0042	0.0423	–0.0167	0.0148	–0.0161	0.0061
4	0.0301	–0.0109	0.0995	0.0241	–0.0807	0.0348	0.0320	–0.0193	0.0784	0.0794
5	–0.0366	0.0338	0.0002	0.0216	0.0519	–0.0237	–0.0316	0.0292	–0.0169	–0.0364
6	–0.0291	–0.0063	0.0520	0.1008	0.0296	0.0201	–0.0232	–0.0057	0.0473	0.1100
7	–0.0447	0.0074	0.1054	–0.0067	–0.0584	0.1151	–0.0400	–0.0050	0.0794	–0.0663
8	0.0533	–0.0156	0.0076	0.0411	–0.0378	0.0634	0.0516	–0.0157	0.0254	0.0796
9	0.0178	0.0556	–0.1124	0.0108	–0.0673	0.0021	0.0141	0.0626	–0.0943	–0.0212
10	–0.0452	0.0579	–0.0178	0.0272	–0.0933	–0.0503	–0.0476	0.0638	–0.0028	0.0744
11	–0.0549	0.0459	0.0036	0.0617	–0.0220	0.0055	–0.0595	0.0498	0.0197	0.1067
12	0.0055	–0.0082	0.0012	0.0445	–0.0377	0.0430	0.0034	–0.0032	0.0283	0.0565
13	–0.0671	0.0627	0.0189	0.0413	0.0685	–0.0119	–0.0667	0.0649	0.0400	–0.0068
14	–0.0589	–0.0080	–0.0711	0.0131	–0.0363	–0.0027	–0.0564	–0.0066	–0.0917	0.0061
15	0.0036	0.0259	0.0601	0.0907	0.0693	–0.0294	0.0050	0.0247	0.0396	0.1658
16	0.0485	–0.0257	0.0627	–0.1236	0.0267	–0.0965	0.0497	–0.0265	0.0578	–0.1267
17	–0.0243	–0.0660	0.0082	0.0611	–0.0050	–0.0484	–0.0257	–0.0613	0.0150	0.0569
18	–0.0562	0.0411	0.0459	0.0532	0.0208	0.0230	–0.0541	0.0321	0.0284	0.0342
19	0.1182	–0.0101	–0.0719	0.1626	0.0216	–0.0868	0.1184	–0.0151	–0.0731	0.1491
20	–0.0134	0.0283	0.0168	0.0064	–0.0758	0.0480	–0.0157	0.0352	0.0407	0.0384

*W*_1*D*_: Columns 71–80										
Row	71	72	73	74	75	76	77	78	79	80
1	0.0704	0.0553	0.0754	–0.0116	–0.0761	–0.0008	0.0423	0.0624	0.0737	–0.0171
2	–0.0057	–0.0899	–0.0688	–0.0199	–0.0459	–0.0480	0.0019	–0.0693	–0.0656	–0.0254
3	0.0957	0.0389	–0.0177	0.0116	–0.0030	0.0243	0.1269	0.0281	–0.0203	0.0111
4	–0.0751	–0.0083	0.0314	–0.0172	0.0569	0.1479	–0.0736	0.0296	0.0311	–0.0179
5	0.0308	–0.0452	–0.0331	0.0288	–0.0364	–0.0285	–0.0031	–0.0749	–0.0299	0.0187
6	0.0034	0.0126	–0.0259	–0.0010	0.0367	0.0870	–0.0167	–0.0143	–0.0220	–0.0022
7	–0.0634	0.0707	–0.0404	–0.0052	0.0550	–0.0911	–0.0200	0.0624	–0.0397	–0.0191
8	–0.0163	0.0761	0.0495	–0.0186	0.0343	0.0549	–0.0269	0.0794	0.0479	–0.0179
9	–0.0029	0.0520	0.0151	0.0610	–0.0703	–0.0805	0.0548	0.0329	0.0133	0.0637
10	–0.0366	–0.0428	–0.0450	0.0610	0.0133	0.0335	–0.0446	–0.0163	–0.0472	0.0682
11	0.0459	0.0058	–0.0578	0.0444	0.0432	0.0650	0.0406	0.0257	–0.0607	0.0506
12	0.0225	0.0571	0.0028	–0.0098	0.0518	–0.0006	–0.0209	0.0588	–0.0017	–0.0040
13	0.1763	0.0054	–0.0652	0.0586	0.0669	0.0024	0.1460	0.0176	–0.0702	0.0615
14	–0.0246	–0.0240	–0.0553	–0.0077	–0.1007	0.0282	–0.0371	–0.0176	–0.0512	–0.0117
15	–0.0397	–0.0246	0.0054	0.0277	0.0323	0.2159	–0.0363	–0.0283	0.0089	0.0184
16	0.0395	–0.1087	0.0471	–0.0328	0.0416	–0.1099	0.0028	–0.1101	0.0508	–0.0398
17	0.0240	–0.0127	–0.0261	–0.0635	0.0255	0.0730	0.0120	–0.0233	–0.0246	–0.0643
18	–0.0177	0.0402	–0.0559	0.0361	0.0110	0.0025	–0.0156	0.0516	–0.0543	0.0301
19	0.0006	–0.0987	0.1150	–0.0125	–0.0872	0.0641	0.0478	–0.1104	0.1134	–0.0209
20	–0.0991	0.0893	–0.0144	0.0323	0.0690	0.0185	–0.0874	0.0543	–0.0144	0.0298

*W*_1*D*_: Columns 81–90										
Row	81	82	83	84	85	86	87	88	89	90
1	–0.0758	–0.0249	0.0721	0.0824	0.0750	–0.0238	–0.0792	–0.0188	0.0532	0.0675
2	–0.0476	–0.0506	0.0404	–0.0360	–0.0668	–0.0216	–0.0540	–0.0963	0.0755	–0.0430
3	–0.0094	0.0389	0.1193	–0.0303	–0.0209	0.0026	–0.0059	0.0642	0.1232	–0.0152
4	0.0562	0.1695	–0.0777	0.0924	0.0295	–0.0026	0.0501	0.2338	0.0007	0.0284
5	–0.0247	0.0096	–0.0367	–0.0178	–0.0288	0.0171	–0.0298	0.0590	–0.0787	–0.0500
6	0.0450	0.0150	–0.0159	0.0037	–0.0189	0.0079	0.0545	–0.0631	–0.0404	–0.0520
7	0.0488	–0.0830	0.1241	0.0987	–0.0402	–0.0205	0.0304	–0.0820	0.2007	0.0596
8	0.0194	–0.0022	0.0172	0.0338	0.0464	–0.0214	0.0254	–0.0500	0.0166	0.0541
9	–0.0779	–0.0994	0.1304	–0.0500	0.0137	0.0559	–0.0653	–0.0942	0.1541	–0.0167
10	0.0074	0.0351	–0.0634	–0.0389	–0.0480	0.0604	0.0126	0.0439	–0.0828	0.0072
11	0.0438	0.0921	–0.0218	0.0066	–0.0618	0.0393	0.0530	0.1277	–0.0702	0.0659
12	0.0445	–0.0475	–0.0658	0.0083	–0.0020	–0.0136	0.0513	–0.0603	–0.1099	0.0528
13	0.0632	0.0131	0.0689	–0.0154	–0.0686	0.0472	0.0651	0.0562	0.0375	–0.0037
14	–0.0967	0.0551	–0.0652	0.0308	–0.0487	–0.0155	–0.1008	–0.0076	–0.0609	–0.0018
15	0.0283	0.2332	0.0253	–0.0184	0.0091	0.0165	0.0221	0.2271	0.0110	–0.0093
16	0.0423	–0.0356	–0.0090	–0.0829	0.0531	–0.0453	0.0448	0.0019	–0.0441	–0.0990
17	0.0129	0.0293	–0.0082	–0.0692	–0.0245	–0.0713	0.0146	–0.0008	–0.0570	–0.0301
18	0.0128	–0.0445	–0.0601	0.1033	–0.0554	0.0390	0.0009	–0.0679	–0.0770	0.0979
19	–0.0999	0.0382	0.0773	–0.1092	0.1096	–0.0188	–0.1056	0.0383	0.1048	–0.1236
20	0.0653	0.0730	–0.0139	–0.0436	–0.0149	0.0128	0.0759	0.1051	–0.0380	–0.0020

## Data Availability

The Open Loop data set of the CSTR setup is available in https://itarasu.com/downloads/

## Abbreviations

CSTR: continuous stirred tank reactor
NARX: nonlinear autoregressive exogeneous
RL: reinforcement learning
MV: manipulated variable
*T*_*r*_: reactor temperature
*T*_*j*_: jacket temperature
